# The influence of reproductive mode on resource competition and diversity patterns in Ediacaran early animal communities

**DOI:** 10.1038/s41559-026-03094-2

**Published:** 2026-06-09

**Authors:** Emily G. Mitchell, Andrea Manica

**Affiliations:** 1 Department of Zoology, University of Cambridge, Downing Street, Cambridge, UK; 2 University Museum of Zoology Cambridge, Downing Street, Cambridge, UK

**Keywords:** Palaeontology, Evolutionary ecology

## Abstract

The appearance of the oldest known animals during the late Ediacaran period (~574 million years ago) was followed by a phase of slow diversification, until a later burst of rapid diversification known as the Ediacaran ‘second wave’. The reasons behind the tempo of diversification are poorly understood. Here we investigate how reproductive mode mediated community dynamics and in turn Ediacaran macroevolutionary change. We show that widespread reproduction via stolon (namely via filaments connecting clones) limited within-species competition, leading to between-species competition acting at smaller spatial scales than within-species competition, a phenomenon called heteromyopia. Heteromyopia enables co-existence of suboptimal competitors because the dispersal limitation of the dominant species limits the occupation of the optimal habitat, so that lesser competitors can still exist within the same community, operating under reduced selection pressure. We explored the consequences of this dispersal limitation on community diversity using a mechanistic model showing that the change from stoloniferous to sexual reproduction that coincided with the second wave could explain the sudden increase in diversity observed in the fossil record. We conclude that widespread asexual reproduction via stolon probably constrained early animal evolution, limiting diversification until the onset of widespread sexual reproduction.

## Abstract (web summary)

The appearance of the first animals in the late Ediacaran period was followed by a period of relatively slow diversification. Here the authors show that asexual stoloniferous reproduction would have enabled co-existence of suboptimal interspecific competitors and constrained diversification.

## Main

**Fig. 1:**
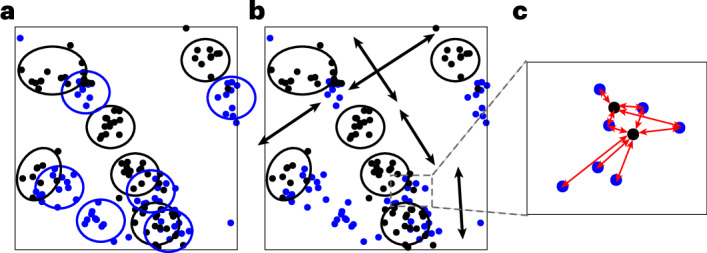
Schematic diagram demonstrating heteromyopia. The blue and black dots represent different species. **a**, If both species are stoloniferous, then all specimens within a cluster (given by the circle) remained connected. **b**, The spatial scales of competition would not be within the cluster (circle), but only between them, shown by the black arrowed lines. **c**, Competition can still occur between two different species, for example, within the grey dashed box in **b**, which would occur on spatial scales smaller than within species where the two clusters overlap (as seen in **a**) and shown by the red arrows in **c**, which depicts the blue specimens that the two black specimens would compete with.

One of the most dramatic events in the evolutionary history of Earth is the appearance of animals in the fossil record during the Ediacaran time period (635–539 million years ago (Ma)), after billions of years of microbial life^1–4^. The earliest widespread animal communities are preserved as Ediacaran macrofossils of Avalonia (574–560 Ma)^3–5^, consisting of in situ, sessile benthic organisms^6–8^. This in situ preservation of sessile benthic communities enables the use of spatial analyses to resolve reproductive modes^9^, habitat associations^10^ and resource competition^10–12^ within Avalonian communities. These spatial analyses have shown that the driving forces behind resource competition are not well understood. Although modern benthic communities are highly competitive, driven by competition for space and/or food (for example, ref. ^13^), such competition is both rare and weak in Avalonian communities^10–12^, with community structure consistent with emergent neutrality^14^. Together, these results suggest that Avalonian systems are compatible with neutral models^12^. However, in Avalonian communities, there are instances of interspecific competition occurring at shorter spatial scales than intraspecific competition^12^, a pattern known as heteromyopia^15^ which suggests that other key processes may be involved^15,16^ (Fig. 1). Importantly, heteromyopic dynamics are hard to distinguish from neutral dynamics based on most ecological metrics, such as species abundance distributions^17^ or spatial point process analyses, the main tools used to show predominately neutral dynamics for Avalonian communities^12^. In extant communities, heteromyopia, although rare^16^, can be caused by dispersal limitation due to connected stoloniferous clusters, pathogens, predators, allopathic death or strong niche segregation^15^. Only by modelling explicitly the spatial scales of interactions, something that has not yet been done for Ediacaran communities, can we directly quantify heteromyopic dynamics, and thus move beyond the niche-neutral paradigm of previous work to explore whether a third process, namely reproductive mode, mediates the community dynamics.

Stoloniferous reproduction^18,19^ within Avalonian communities was first inferred on the basis of the size-structured spatial arrangement of specimens^9^, with further evidence provided by numerical simulations and computational fluid dynamics^20^. In terms of physically preserved stolon, filaments have been found connected to five Avalonian taxa^18,19^, suggesting preserved stolon, although non-stoloniferous filaments are also probably present^21,22^. Stoloniferous reproduction has the capacity to reduce intraspecific competition as follows: if stoloniferous clones remain connected by stolon throughout their lives, they would share nutrients (regardless of feeding mode or height of feeding) between these clones, thus acting as a unit and negating the need for nutrient competition between individuals (Fig. 1a). If one clone is in a low resource area, it can gain nutrients via stolon from clones in higher resource areas. As such, competition will not occur within connected stoloniferous networks, it would only occur between clonal colonies since each connected clonal colony is acting as a unit (Fig. 1b). Therefore, stoloniferous reproduction could be a mechanism for the relatively low levels of intraspecific competition and heteromyopia that is found within Avalonian communities^10,11^ which has the potential to lead to low alpha diversity (species richness) levels^23^.

In this study we test the importance of intraspecific processes, specifically stoloniferous reproduction, to the presence and strength of intraspecific resource competition (that is, quantify the strength of heteromyopic dynamics) and use mechanistic models to investigate how these processes may have impacted the biodiversity of early animal communities of the Ediacaran (~574–539 Ma).

## Identification and quantification of stoloniferous reproduction

**Fig. 2:**
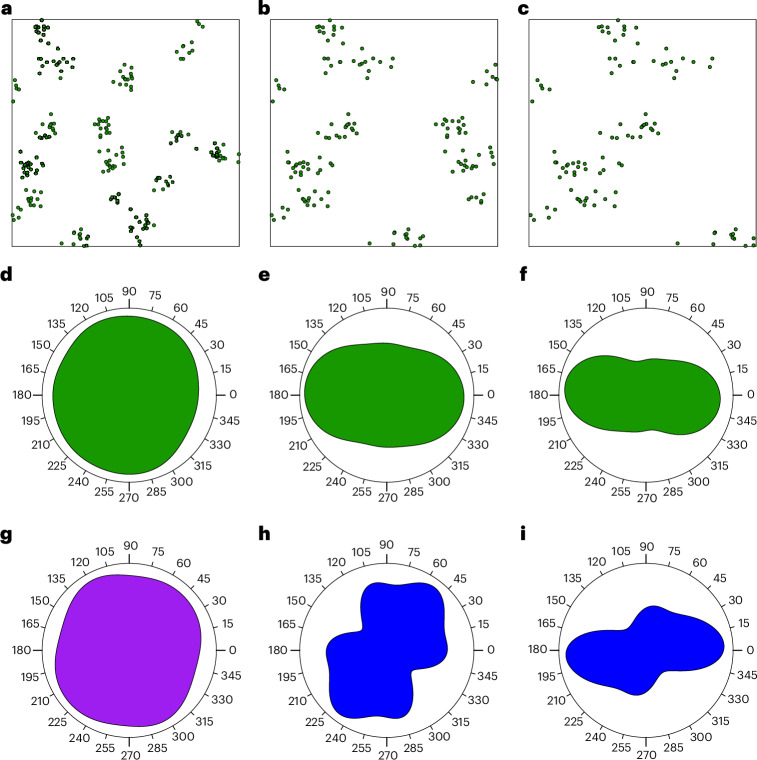
Examples of different levels of isotropy. **a**–**i**, Simulated Thomas clusters (**a**–**c**) with their associated rose diagram (as one would expect for clonal organisms) (**d**–**f**)**. b**, The same Thomas clusters as **a**, with 2× directionality on the *x* axis (as might be caused by broadcast spawning). **c**, The same Thomas clusters as **a**, with 3× directionality. **d**–**f**, Rose diagrams for Thomas clusters, with angles in degrees, are given in **a** for simulated Thomas Clusters with no directionality, so with corresponding IQR = 0.0006. Rose diagram for **b** with2× directionality is given in **d**, with IQR = 0.0007, and in **e** with 3× directionalitywith IQR = 0.0011 (**f**). **g**, The isotropy of observed examples of a hydroid colony connected by stolon, with IQR = 0.0001. **h**,**i**, The deep-sea sponge *A. magnifica* in two sections of a deep-sea coral and sponge community subject to different current directions, with IQR = 0.0023 (**h**) and IQR = 0.0014 (**i**) (from ref. ^72^).

Stoloniferous reproduction restricts the direction and distance offspring can travel from their parents, as the children remain attached via these stolon to their parents. This attachment results in isotropic clusters, that is clusters that do not have any directionality (are circular). Stoloniferous reproduction also results in small clusters sizes because the offspring cannot be transported long distances by currents (as per refs. ^9,20^; Fig. 2a,d,g). On the other hand, where benthic organisms disperse pelagically transported by currents (either via pelagic larvae or pelagic buds or fragments), we expect clusters to be elongated, following the direction of the current (Fig. 2b,c,e,f,h,i). For a signal of stoloniferous reproduction to be found, the population should also be sufficiently mature to have reached reproductive age, so the relative age of the population may also impact the ability to find such reproductive modes^9^.

We used the interquartile range (IQR) of all point-to-point orientations within a population to quantify cluster directionality where the point-to-point orientations are the orientation between each point (fossil specimen) to all the other points (conspecifics fossil specimens) within the population. This IQR is visualized by plotting the point-to-point orientation measurements as rose diagrams, with isotropy indicated by small values (round rose diagrams; Fig. 2d,g) and anisotropy by high values (ovate rose diagrams; Fig. 2e,f,h,i).

There are three factors that can impact the size of dispersal clusters. First, stoloniferous connection between individuals will limit the size of dispersal clusters, since waterborne propagules travel further than connected ones^24–27^, leading to smaller cluster sizes. Second, because such reproductive cluster sizes depend on the height of the parent, here we consider normalized clusters (that is, we divide the cluster size by the maximum specimen height of population) rather than absolute sizes. The third factor that would influence the prevalence of stoloniferous reproduction is the relative maturity of the population. Well-established populations will have had more time to reproduce, so are likely to have higher percentages of the second/third generations. If new generations result from stoloniferous reproduction, then we would expect to see stronger effects of stoloniferous reproduction for more mature communities. Therefore, as a proxy for relative age of a population, we used the percentage of the population that occupies the smallest size class, with the larger the percentage, the younger the population.

## Identification and quantification of resource competition

Crucial to determining the relationship between reproductive mode and community dynamics is determining the strength of competition within these communities. Although identifying the most likely process underlying a spatial pattern is not straightforward^28–32^, competition within sessile communities can be identified through spatial regularity (intraspecific) and segregation (interspecific) as the most likely underlying process which is not linked to underlying habitat associations^33,34^. The presence of resource competition has previously been detected within Avalonian communities using spatial point process analyses (SPPA)^9,11,12^, identifying four cases of interspecific competition within seven communities. However, these studies focussed on detecting competition, rather than estimating its strength^9,12,35^. Here we use SPPA to quantify competition strength and use this metric to investigate its drivers. SPPA can be used to quantify the level of spatial regularity and segregation that is not caused by habitat association^33,34,36^: specifically, we use the distance measure pair correlation functions (PCF) to quantify how the density of points (fossil specimens) changes across the mapped area (bedding planes)^37^ of 21 populations across 8 bedding planes from the Avalon of Newfoundland, Canada, and Charnwood Forest, UK, using pre-existing data^9,12,35,37^. We use univariate PCF for intraspecific analyses, where PCF = 1 represents complete spatial randomness (CSR), with values below 1 corresponding to spatial regularity and above 1 to spatial aggregation. For the interspecific analyses, PCF < 1 correspond to spatial segregation and PCF > 1 to positive spatial association. The strength of regularity/segregation, that is resource competition, is indicated by the smallest PCF value, that is the minimum value of the PCF and the spatial scale over which resource competition occurs is the regularity/segregation up to (or over) the point where the observed data cross PCF = 1 (ref. ^37^).

## Results and discussion

### Relationship of reproductive mode to resource competition

**Fig. 3:**
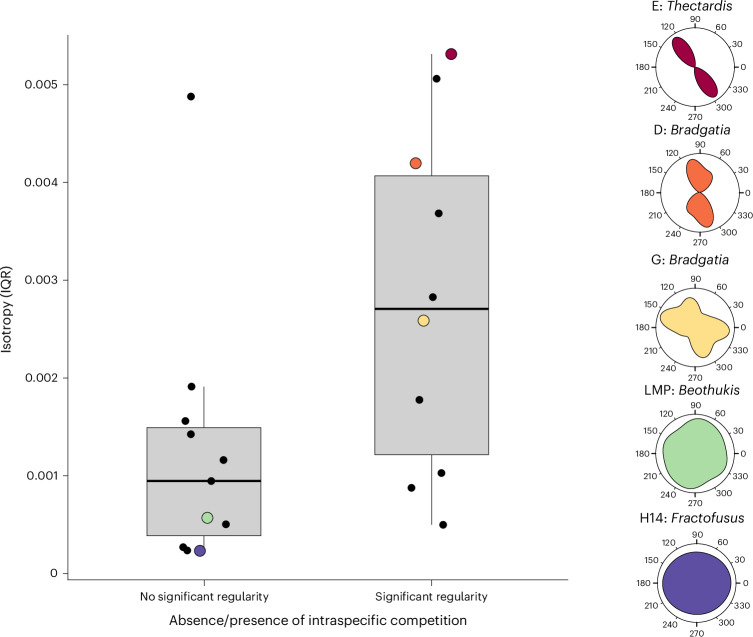
Relationship of isotropy to intra-specific segregatiom. Isotropy is measured by the IQR of point-to-point orientations grouped depending on whether they exhibit significant regularity: lower IQR indicates isotropy and higher IQR anisotropy. To visualize how the IQR corresponds to directionality, rose diagrams for five example taxa are given on the right, with the corresponding IQR given on the boxplot in the same colour. The median percentile is shown, with boxes providing the lower (25th) and upper (75th) quartiles and whiskers 1.5× IQR. Median isotropy for non-regular taxa was 0.0009 and for regular spaced taxa was 0.0027. Sample sizes are provided in Extended Data Table 1. We found an association between the IQR of cluster directionality (isotropy) and presence/absence of intraspecific regularity (two-sided Mann–Whitney *W* = 25, *N*_1_ = 10, *N*_2_ = 11, *P* = 0.036).

We started by applying our new directionality measure IQR to 21 taxa populations (data from ref. ^12^). There was a range of cluster directionality as indicated by the IQR of point-to-point orientations (Fig. 3 and Extended Data Table 1), with H14 *Fractofusus andersoni* showing the highest levels of isotropy and *Thectardis* on E surface showing the lowest levels of isotropy (IQR_H14: *Fractofusus*_ = 0.0002; IQR_E: *Thectardis*_ = 0.0053; Fig. 3 and Extended Data Table 1). The *F. andersoni* result is consistent with previous work that used spatial analyses to infer stoloniferous reproduction^9^, as well as physical evidence of filaments connected to specimens^18,19^. By contrast, the Bristy Cove *Fractofusus* showed high levels of directionality, probably due to the relative immaturity of the community (as inferred by the small body sizes and large percentage in the smallest size class; Extended Data Table 2), suggesting that the population lacked sufficient time to reproduce^18,38^. The breadth of directionality results (Extended Data Table 1) demonstrates that stoloniferous reproduction was occurring in varying amounts across and within several bedding planes, enabling further analyses to be able to test the consequences of such reproductive mode.

**Extended Data Fig. 1:**
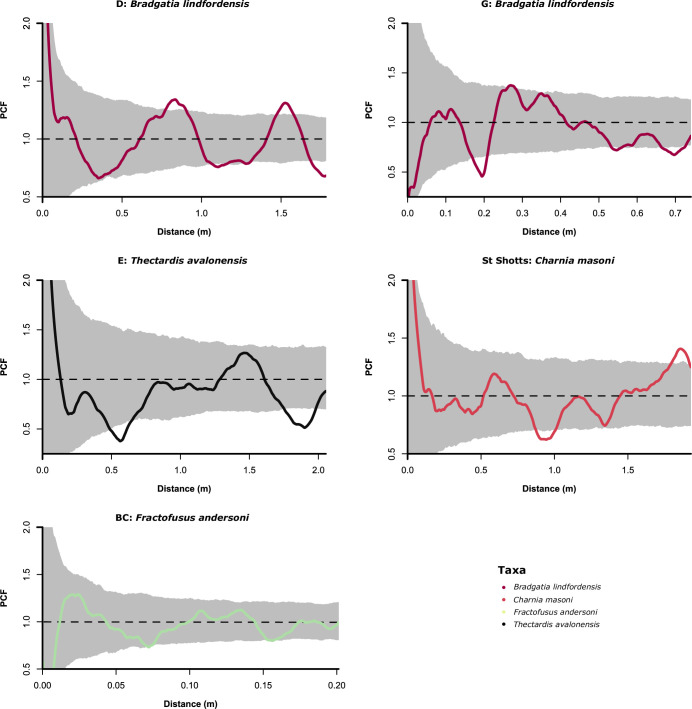
PCF for mapped taxa that exhibit inter-specific regularity but no bivariate segregation. For all plots the x-axis is the inter-point distance between organisms in meters. The y-axis PCF = 1 indicate CSR, <1 indicates regularity, and >1 indicates positive association. Grey shaded area depicts the bounds of 999 Monte Carlo simulations of CSR, with non-CSR patterns indicated by PCF curves are not completely within these areas. The solid black line is the observed PCF. Sample sizes are provided in Extended Data Table 1.

**Extended Data Fig. 2:**
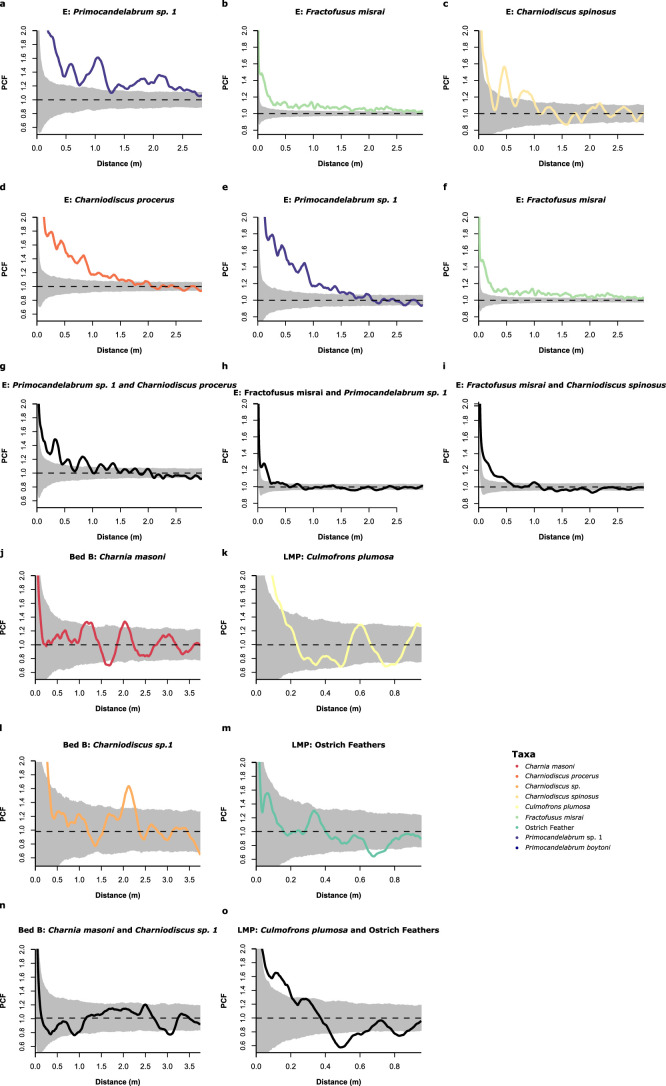
PCF for mapped taxa pairs that exhibit inter-specific segregation. For all plots the x-axis is the inter-point distance between organisms in meters. The y-axis PCF = 1 indicate CSR, <1 indicates regularity, and >1 indicates aggregation. Grey shaded area depicts the bounds of 999 Monte Carlo simulations of CSR, with non-CSR patterns indicated by PCF curves are not completely within these areas. The solid black line is the observed PCF. Sample sizes are provided in Extended Data Table 1. **a** and **d** are the univariate PCFs for bivariate PCF in **g.** **b** and **e** are the univariate PCFs for bivariate PCF in **h**. **c** and **f** are the univariate PCFs for bivariate PCF in **i**. **j** and **i** are the univariate PCFs for bivariate PCF in **n**. **k** and **m** are the univariate PCFs for bivariate PCF in **o**.

We quantified the strength and spatial scale of intraspecific competition, using the approach developed previously^9^. There are ten taxa populations which had excursions under the Monte Carlo simulation envelopes that were not best modelled by heterogeneous Poisson models (which would indicate effects of habitat associations; compare ref. ^10^), so were considered to exhibit intraspecific competition (Extended Data Figs. 1 and 2 and Extended Data Table 1). G surface *Bradgatia* exhibited the strongest regularity (PCF_min_ = 0.6151; Extended Data Fig. 1). E surface *Charniodiscus procerus* had the weakest of the significant regularities (PCF_min_ = 0. 9252; Extended Data Fig. 2), with a median PCF_min_ = 0.6732 for the populations that exhibited intraspecific spatial regularity (Extended Data Table 1) and median PCF_min_ = 0.9463 for those that did not.

**Fig. 4:**
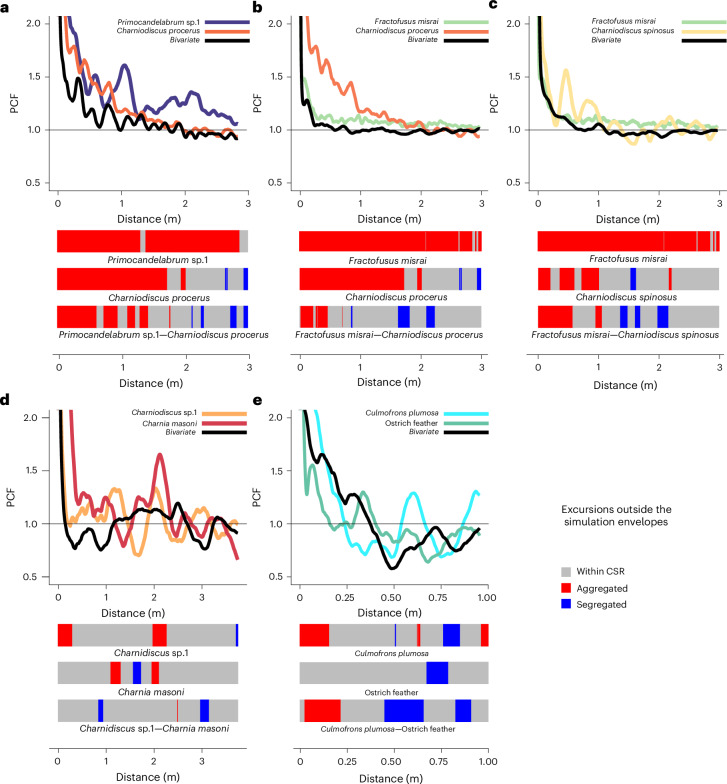
PCF for mapped taxa pairs that exhibit interspecific segregation. For all plots, the *x* axis is the interpoint distance between organisms in metres. The *y* axis PCF = 1 indicate CSR, <1 indicates segregation and >1 indicates positive association with the bars below the plots indicating whether the PCF is significantly >1 (red), <1 (blue) or CSR (grey). Heteromyopia is where the segregation (blue in the bottom bar) occurs at a smaller spatial scale (smaller *x* axis value) in the interspecific bar than in the univariate bars. **a**, The spatial patterns for E surface *Primocandelabrum* sp1. and *C. procerus*. **b**, The spatial patterns for E surface *Fractofusus misrai* and *C. procerus*. **c**, The spatial patterns for E surface *F. misrai* and *C. spinosus*. **d**, The spatial patterns for bed B *Charniodiscus* sp1. and *C. masoni*. **e**, The spatial patterns for lower Mistaken Point surface *Culmofrons plumosa* and Ostrich feather.

There were five cases of significant interspecific segregation on smaller spatial scales to intraspecific segregation (Fig. 4). There were three interspecific distributions where the spatial scale of the minimum PCF was smaller than for two species and two where it was smaller than both species (Fig. 4). The largest heteromyopic difference of spatial scales was with *Fractofusus* and *C. procerus* on E surface, where the interspecific segregation occurred 1.21 m before the intraspecific regularity of *C. procerus* and 2.85 m before *Fractofusus* (Fig. 4). Resource competition can be detected on large spatial scales, such as here, when such competition leads to spatial thinning whereby the communal resources are reduced by specimens, leading to a specimen density reduction throughout the population(s). These results demonstrate the heteromyopia present across these communities (Fig. 4).

### Relationship between stoloniferous reproduction and strength of intraspecific regularity

**Fig. 5:**
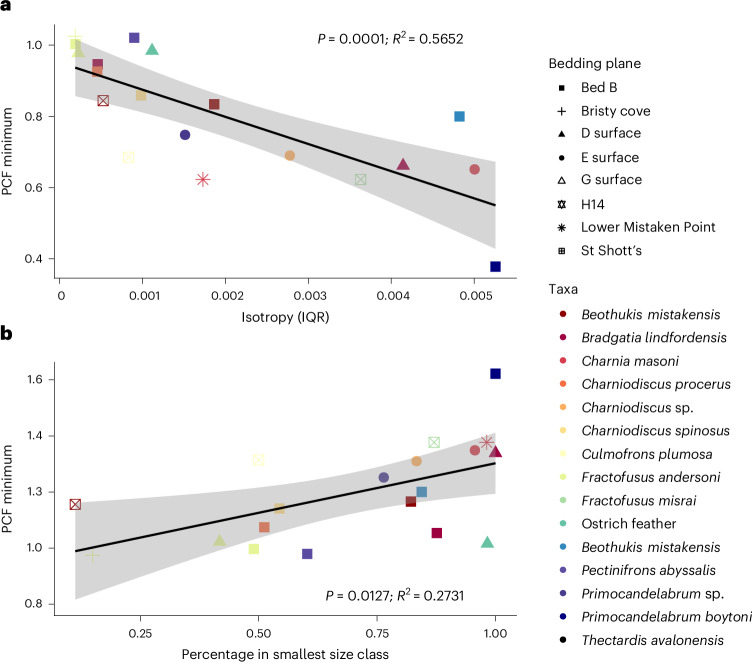
Relationship between predictive variables with the most power and the minimum PCF. Black line shows the best fit for each regression and the grey area is the 95% confidence interval. **a**,**b**, The regression between the IQR (cluster directionality) and the minimum PCF (**a**) and between the percentage in the smallest size class (proxy for population age) and the minimum PCF (**b**).

Having established a measure of stoloniferous reproduction (IQR of cluster directionality; Fig. 2) and a measure of the strength of intraspecific regularity (Extended Data Fig. 2 and Extended Data Table 1), we can systemically test for an association between the two. We found an association between the IQR of cluster directionality (isotropy) and presence/absence of intraspecific regularity (two-sided Mann–Whitney *W* = 25, *N*_1_ = 10, *N*_2_ = 11, *P* = 0.036) (Fig. 3), suggesting that stoloniferous reproduction inhibits the presence of intraspecific competition. Furthermore, we found that the strength of intraspecific regularity was best predicted by IQR (cluster directionality) (Fig. 5 and Extended Data Table 3; *F*_1,17_ = 24.4, *P* « 0.0001, adjusted *R*^2^ = 0.5621) with model with combination cluster directionality and percentage of the population in the smallest size class was also a good model fit (Fig. 5 and Extended Data Table 3; *F*_2,16_ = 11.61, *P* = 0.0008, adjusted *R*^2^ = 0.5411, Akaike information criterion (ΔAIC) = 1.87).

Our results were robust to variations in how the PCF was estimated, namely the spatial scales, smoothing factors and when we only include regular spaced populations in our regressions (Extended Data Table 4). For the best-fit model, all permutations had *P* < 0.05. These regressions and associated sensitivity analyses support the hypothesis that the strength of intraspecific competition is reduced within stoloniferous populations.

To consider the most likely mechanism behind heteromyopia, we need to consider how each of the five processes that can lead to heteromyopia, namely stoloniferous clusters, pathogens, predators, allopathic death or strong niche segregation^15^, could manifest themselves within these Avalonian communities. Within Avalonian communities, there is no evidence of macropredation until the terminal Ediacaran, making predation unlikely. Niche influences on Avalonian taxa are rare and weak^10,12^ and there is no evidence of Avalonian niche segregation^39^, so niche segregation is also unlikely. The highly repetitive nature of Avalonian spatial patterns across potentially large space and time intervals^12^ also makes niche processes less likely. Allelopathy and pathogens would be expected to have stronger effects at the smallest spatial scales, because the chemicals (or pathogens) have the highest density around the individual, so would lead to the largest regularity at small spatial scales, which decrease as spatial scale increases. Such strong, small-scale regularity is not observed in any of the eight communities (Extended Data Figs. 1 and 2). Of the eight communities, only four had sufficiently abundant multitaxa populations to enable bivariate analyses. Of these four, three communities had non-random spatial distributions (Fig. 4). Within these three communities, there are five instances of bivariate distributions where the spatial scale of interspecific competition occurs at smaller spatial scales than intraspecific competition at least one taxon (Fig. 4), indicating heteromyopia; that is all three communities that had non-random bivariate spatial patterns exhibited heteromyopia^10,12,15,39^. Because there is a significant association between factors associated with stoloniferous reproduction and resource competition (indicated by spatial regularity), we suggest that connected stoloniferous networks probably drive the heteromyopic patterns found.

### Consequences of dispersal limitation on Ediacaran diversification patterns

Our data are a good representation of Avalonian life because the communities we studied include 76% of documented species from the Avalon^40^, covering 572–560 Ma. In this study, we analysed all the abundant populations, which accounted for 46% of documented species with a further 30% of species found within the mapped areas but not in sufficient abundances for these analyses (*n* > 30) and the remaining 26% of taxa considered rare (<10 documented specimens)^40,41^. Within our studied communities, where resource competition occurs (indicated by spatial segregation), heteromyopia occurs in three of the four communities sufficiently abundant for bivariate analyses, including between the most abundant species populations, indicating extreme dispersal limitation, so is probably a dominant force behind community dynamics.

Dispersal limitation changes the dynamics of communities in terms of selection pressures on good and bad competitors^15^. In non-dispersal limited communities, the strongest competitor will take over all the optimal habitat, leading to competition–colonization trade-offs, strong selection pressures and the best competitors leaving little room for suboptimal competitors to establish themselves^42^. However, where dispersal is strongly limited, such as for connected stoloniferous networks, populations occupy a mixture of good and bad resources, as long as at least some of the connected stoloniferous network is in the good habitat^43,44^. Unlike dispersal unlimited taxa, which are able to spread widely and colonize all the optimal resources, dispersal limited taxa cannot spread so easily, so often optimal resources are still available to suboptimal competitors^45^. This dynamic creates a mechanism of co-existence whereby weaker competitors co-exist alongside much stronger competitor taxa^45^ and so are under reduced selection pressure^15,46^.

Our results thus suggest that in these Avalonian communities, stoloniferous reproduction can lead to heteromyopia. Yet, most taxa (70%) are not abundant, so do not form a substantial proportion of the communities and are not subject to these dispersal limited processes; instead, they enjoy an easier existence, with reduced selection pressure. This dispersal limited dynamic is consistent with the accumulation mode of community development^14^. In such communities, instead of communities developing systematically in response to a deterministic set of interspecific interactions, the compositions of the communities do not change through time and are dependent on the initial colonizers, whose reproductive mode shapes how the communities mature.

The lack of selection pressure for most Avalonian taxa (owing to this dispersal limitation) may explain the relative low rates of Avalonian diversification, with most taxa not under strong evolutionary pressure, but the dominant taxa exerting a dispersal limited dynamic, thus enabling co-existence without a strong driver for natural selection. This co-existence dynamic would destabilize if there was a change in dominant reproductive strategy, from stoloniferous to waterborne, that is sexual reproduction (compare ref. ^47^). Facultative sexual reproduction is common in extant sponges^48^ and cnidarians^49^ and also inferred in Avalonian taxa, for example with *Fractofusus*^9,50^ and *Charnia*^18,51^. In extant animals, the relative dominance of asexual to sexual reproduction can change depending on the environment, with less stable, more disturbed habitats triggering higher levels of sexual reproduction^52^.

The deep-sea Avalonian environment was relatively stable compared with the White Sea shallow water environment, with a more homogeneous background environment^53^ and the death-events that killed and preserved the communities occurring around every 10–100 years, possibly up to every 1,000 years^54–56^, rather than much more frequently, two or three times per year, in the White Sea environment^57,58^. This less-disturbed Avalonian environment is evidenced by a greater proportion of mature communities^14^ and the older inferred maximum ages of specimens^59^. Therefore, the colonization of the more disturbed shallow water environment may have triggered a switch from asexual dominated communities to sexual dominated communities. The increase in sexual (that is, waterborne) reproduction, and its associated reduction in dispersal limitation^60–63^, increases competition and selection pressure for both the previously dispersal limited animals as well as the weaker competitors that had previously been shielded by the dispersal limited dominant taxa. Thus, increase in Ediacaran diversity around 550 Ma (ref. ^64^) has the potential to be explained from the increase of dispersal distances^65^ and subsequent changes of selection pressure that comes with a transition from widespread stoloniferous reproduction, to systems dominated by waterborne propagation^47,65^.

### A mechanistic model to investigate the effect of dispersal limitation

To investigate the impact of dispersal limitation on alpha diversity we constructed a mechanistic model of the Avalon, White Sea and Nama assemblages (compare ref. ^66^). These three assemblages represent the taxonomic grouping of Ediacaran macrofossil taxa, with each assemblage occupying largely different temporal periods and different environmental settings^40,67^, and they are often used when discussing Ediacaran evolutionary patterns to distinguish different evolutionary phases. These three assemblages show a dramatic increase in alpha diversity between the Avalon and White Sea assemblages, with a more limited decrease in the Nama^68^. To see the extent to which dispersal limitation can explain these trends we created a mechanistic model to simulate alpha diversity. Our model was a spatially explicit lottery metacommunity model^66^, parameterized by the extent of immigration between communities, spatial autocorrelation (that is, the extent of association with habitat heterogeneities), distance of dispersal and niche overlap. In this mechanistic model, dispersal was modelled as the relative percentage of specimens after a reproductive event that left the local community, that is joined the rest of the metacommunity, such as where dispersal parameter *ω* < 1.0, 100% of new specimens would remain inside the community and *ω* > 1.0 reflects where specimens emigrate. As such, *ω* represents the degree of dispersal limitation. For example, if all specimens were reproducing via stolon, we would expect *ω* *«* 1.0 since none would be waterborne, so they would not be able to leave their community. By contrast, if all specimens were reproducing via spawning, they would be carried in the water, so a large proportion would leave their natal community. Within this model, heteromyopia is a consequence of high dispersal limitation.

**Fig. 6:**
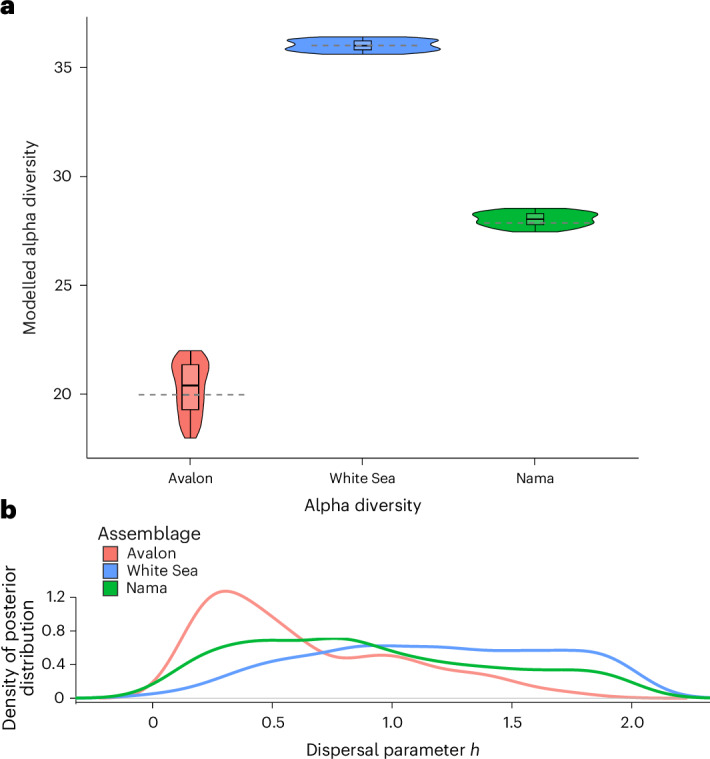
Mechanistic model simulations with rejection sampling using ABCs with neural networks. **a**, Resulting alpha diversity from the 10,000 model simulations fitted to the observed (sampling corrected) alpha diversity for the Avalon, White Sea and Nama assemblages. The grey dashed lines indicate the observed alpha diversity for the three assemblages whereby database occurrences were randomly subsampled to 50 occurrences from ref. ^23^. The median percentile is shown, with boxes providing the lower (25th) and upper (75th) quartiles and whiskers 1.5× IQR. **b**, The posterior distribution for the dispersal parameter for the three assemblages, with low dispersal in the Avalon and high dispersal in the White Sea and Nama assemblages^12,39,73^.

**Extended Data Fig. 3:**
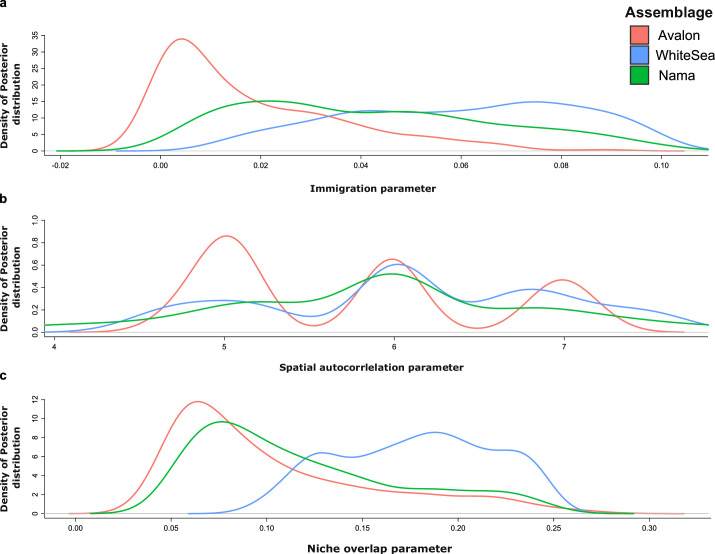
The posterior distributions for the a) immigration parameter, b) spatial autocorrelation parameter and c) the niche overlap parameter. **a**) shows increasing immigration (although still low) between the Avalon and White Sea assemblages, with medium values for Nama. **b**) Shows the three discreet values for spatial autocorrelation (5, 6, and 7), with the Avalon showing a clear distinction of low spatial autocorrelation, with more similar values for the White Sea and Nama assemblages. **c**) shows high levels of niche overlap for the Avalon with medium to low niche overlap for the White Sea assemblage. Nama assemblage shows lower, but similar levels of niche overlap to the Avalon assemblage.

Given the observed values of alpha diversity for the Avalon, White Sea and Nama assemblages^23^, we found the posterior distributions of the dispersal parameter from its full range using approximate Bayesian computation (ABC)^69^ using single-hidden-layer neural networks^70^. Our mechanistic model was able to reproduce the observed alpha diversity levels (Fig. 6a). Although the posteriors were broad (as one would expect given the limited number of available communities) the shape of the dispersal distribution differs among the three assemblages, showing a clear increase from the Avalon to the White Sea and a decrease in the Nama (Fig. 6, Extended Data Table 5 and Extended Data Fig. 3 for other posterior distributions). If dispersal did not have a role in the increased diversity, we would expect the posterior for that parameter not to differ among the three periods. Therefore, the shape of the posteriors we obtained demonstrates that dispersal increase can explain the observed patterns of Ediacaran species richness^23,64,71^ (Fig. 6). Our mechanistic model fitted by ABC seems to capture the major trends in speciation experienced through the Ediacaran, suggesting that the dispersal limitation of stoloniferous reproduction is sufficient to explain the increased diversity measured between the Avalon and White Sea assemblage.

Our results have shown that stoloniferous reproduction in the Avalon assemblage has a significant relationship with the presence and strength of intraspecific competition. We have further demonstrated that the dispersal limitation induced by stoloniferous reproduction is the most likely underlying source of Avalonian heteromyopia, thus reducing selection pressure, with our mechanistic model indicating that these effects of stoloniferous reproduction are sufficient to explain the key trends of Ediacaran diversification. As such, stoloniferous reproduction probably had a key role in constraining early animal evolution, reducing diversification. The later onset of mobility and widespread sexual reproduction, and the consequent reduction of stoloniferous reproduction, led to an increase in competition, shifting the communities to a more niche-based dynamics^12,23,39,72,73^.

## Methods

### Quantification of isotropy of taxon populations

Isotropy (directionality of clusters) of the spatial distributions was quantified using the spatstat package^74^ in R, by calculating the IQR of the orientations between every point within a population and every other point within a given radius^75^. These data are plotted as rose diagrams (Fig. 3 and examples in Fig. 2), where isotropy is indicated by only small variations (that is, IQR) in the orientations, namely a relatively circular rose diagram. Strongly anisotropic distributions are highly non-circular, that is exhibit large variations so ovate rose diagrams. This variation is captured using the IQR, where large values indicate high levels of anisotropy and low values indicate isotropy. To illustrate how IQR of point-to-point orientations captures directionality we have run our analyses on spatial data from three simulated examples. We first simulated Thomas cluster distributions (Fig. 2a), with no inherent directionality, which showed a relatively isotropic, namely circular rose diagram (Fig. 2d). We then applied a 2× transformation to these Thomas cluster points along the *x* axis only (Fig. 2b), which showed an ovate rose diagram (Fig. 2e), with a 3× transformation (Fig. 2c), showing a more elongated rose plot (Fig. 2f). To illustrate how IQR varies with different reproductive modes, we ran our analyses on three observed examples. First, an extant hydroid stoloniferous colony^76^ to demonstrate the high isotropy of stoloniferous reproduction (Fig. 2g) and then for the sponge *Advhena magnifica* in two areas of an extant deep-sea coral and sponge community^72^ exhibiting high anisotropy, with the anisotropy varying as a result of different current direction on the two different areas (Fig. 2h,i).

### Intraspecific and interspecific species spatial distributions

The data used in this study have already been published^12,35^ and so some SPPA have already been performed on these data as follows:

- D and E surface: intraspecific spatial distributions up to 0.5 m (ref. ^9^) and 2.0 m (ref. ^12^); E surface interspecific up to 4.5 m (ref. ^11^)
- LMP interspecific up to 1.0 m (ref. ^10^)
- Bed B: intraspecific and interspecific up to 0.5 m (ref. ^12^)
- H14: intraspecific spatial distributions up to 0.5 m (ref. ^9^) and 2.0 m (ref. ^11^)
- Bristy Cove: intraspecific spatial distributions up to 0.25 m (ref. ^11^)
- St Shotts: intraspecific spatial distributions up to 1.5 m (ref. ^11^)

This published work is extended in this study by (1) extending the spatial scales of the Bed B analyses from 0.5 m to 3.5 m; (2) analysing the intraspecific spatial distributions from LMP and (3) performing new intraspecific analyses on the G surface *Bradgatia* population (data in ref. ^35^). The previous SPPA was published across four different studies^9–12^, so to ensure that the data were analysed consistently, all intraspecific and interspecific spatial distributions were (re)analysed as described below. These SPPA provided the data needed for the regression analyses, namely the presence or absence of aggregations/associations and regularities/segregations, the regularity strength as measured by the value of minimum PCF values and the size of cluster radius.

For all 21 populations the density, maximum specimen height was recorded (Extended Data Table 1) and number of normally distributed cohorts within the size distribution of the populations was found^77^ by fitting height–frequency distribution to various models, followed by comparison of (logarithmically scaled) Bayesian information criterion (BIC) values, which we performed in R using the package MCLUST^77^ (Extended Data Table 2). A BIC value difference of >10 corresponds to a ‘decisive’ rejection of the hypothesis that two models are the same, whereas values <6 indicate only weakly reject similarity of the models^78^. For each population the percentage within each size class is recorded to indicate the number of juveniles within each population.

To extract the spatial scale of aggregation/associations and/or spatial regularity/segregation, spatial analyses are performed in R using the package spatstat^74^ (described in detail in ref. ^79^). These spatial patterns are used to extract not only the PCFs for spatial scale and strength of aggregations/associations and/or regularity, but also the IQR, background heterogeneity (LH*) and the size of aggregation clusters (Extended Data Table 1) as predictor variables for the regressions.

The mapped areas are irregularly shaped, so to account for edge effects, Ripley’s isotropic edge correction weight is used^80^, whereby the number of points within an incomplete circle are scaled by the proportion of the area of the circle that lies within the mapped area^79^. To quantify the spatial distributions and find any significant deviations from CSR, PCF are used^37^. The spatial scales and magnitudes of non-random spatial patterns are determined by running 999 Monte Carlo simulations for each species, on a homogeneous background, to generate simulation envelopes around the random (PCF = 1). Homogeneous backgrounds were used because previous work has established the lack of significant influences by heterogeneous backgrounds for these Avalonian communities^12^, so such heterogeneities do not need to be taken into account, in contrast to many modern systems^33,81^. The highest and lowest 5% of simulations are excluded from the simulation envelope to exclude outliers^79^. Where regularity (PCF < 1) fell outside the Monte Carlo envelope, the minimum PCF value is recorded. So that larger values indicate more regularity^37^, the metric (1 − PCF_Min_) is used.

Reproductive processes can be modelled spatially using Thomas cluster models^9,31,37,79^, which are parametrized by the number of clusters in a given area (the density of the clusters), the density of points (specimens) within a cluster and the radius of the clusters. The density of the points within the cluster follows a Gaussian distribution such that the highest density is in the centre of the cluster, that is around the presumed parent. The number of ‘offspring’ in a cluster is therefore a function of the density within the cluster and the size of the cluster. Thomas cluster models are fitted to the small-scale PCF to determine the best-fit cluster size for the distribution^74,82–84^. Thomas clusters^9,31,37,79^ have been established to be the dominant mode of aggregation for Avalonian communities^12^, so are appropriate for this modelling.

To compare how variables associated with reproduction differ in influence on resource competition, we also calculate the LH* metric which quantifies the degree of patchiness within different systems^85^. The LH* is calculated by estimating how much nearest-neighbour distances between the fossil specimens vary compared with expected random nearest-neighbour distributions (modelled using a homogeneous Poisson model)^85^, with high values indicating greater levels of heterogeneity.

### Testing for associations between the presence and strength of intraspecific competition and stoloniferous variables

The presence of intraspecific competition is indicated by an excursion below the Monte Carlo simulation and tested against isotropy (IQR), the relative maturity of the population (percentage of specimens in the smallest size class), environmental heterogeneity (as indicated by LH*) and the normalized cluster size (Extended Data Table 1). Model fit is compared using AIC in a stepwise approach^86^, with the best-fit model reported (Extended Data Table 3). The strength of intraspecific competition (as indicated by the minimum PCF value of an excursion below the Monte Carlo simulation) is tested against the population metrics (Extended Data Table 1).

### Sensitivity analyses

In terms of the input variables for our regression, there are three different sets of permutations that can impact the PCF and so influence the minimum PCF value as follows. First, the range of PCF distances (*r*) because the calculated PCFs near *r* = 0 and near the edge of the distance range can be unstable. Second, the PCF is calculated using a smoothing estimator to account for noisy data. We have used as default the kernel bandwidth $\frac{c}{\sqrt{\lambda }}$ where *λ* is the point density and *c* is between 0.1 and 0.2 (refs. ^75,84^), with the default in spatstat set to *c* = 0.15 (refs. ^75,84^). The larger the bandwidth, the more smoothing is applied, so the signal is less noisy. Third, whether we consider all spatial patterns or only the ones that have PCF < 1, that is exhibit some spatial regularity. To test the impact of changing these parameters on our regressions we calculated 24 different permutations for the PCF to find the minimum PCF as follows: four sets of different distance ranges. (1) All distances, (2) excluding the very small (<1% of maximum distance), (3) excluding the small distances that probably overlap with the body sizes of the specimens (~20 cm <5%), and (4) excluding the small and very large (5% < *r* < 95%). For each distance permutation we calculated the PCF for three different levels of bandwidth: (1) less smooth (*c* = 0.1); (2) default/medium (*c* = 0.15), and (3) smoother (*c* = 0.2). Then for each permutation of distance and smoothing we input (1) all the populations and (2) only the populations that exhibited PCF < 1 over the distance ranges and smoothing considered.

### Mechanistic models with ABC alpha diversity

To explore how dispersal limitation, immigration, niche overlap and environmental heterogeneity impacts alpha diversity, we use the metacommunity model of ref. ^66^ describing 100 communities on a grid network. This model is a lottery model with discrete time steps, which has been used to focus on sessile marine organisms to assess the impact of dispersal distances, niche overlap and spatial autocorrelation on biodiversity patterns. Within this mechanistic model framework, a reproductive event followed by dispersal are simulated, then the alpha diversity across the total number of communities is calculated. Full mathematical details are described previously^66^. Qualitatively, the reproduction rate depends on the niche overlap and the environmental condition (parameterized between zero and one for each community) within a community. The niche overlap *h* is the variance of the Gaussian distribution that describes how much a given species overlaps with another, such as high *h* corresponds to more niche overlap and low *h* to a more specialized niche. Dispersal is assumed to be passive (such as through corals spawning), with the dispersal parameter as the relative percentage of specimens after a simulated reproductive and then dispersal event than remain within the community, versus leave the local community and so join the rest of the metacommunity. Where dispersal parameter *ω* < 1.0, all new individuals remain inside the community and *ω* > 1.0 reflects where individuals leave the local community and join the rest of the metacommunity. For example, if all specimens were reproducing via stolon, we would expect *ω* *«* 1.0 since none would be waterborne, so they would not be able to leave their community. By contrast, if all specimens were reproducing via spawning, they would be carried in the water, so the majority would leave their natal community and join the wider metacommunity.

Spatial autocorrelation defined as high autocorrelation reflecting communities that are closer together have similar environmental conditions, whereas low spatial autocorrelation means that the environmental conditions of nearby communities are unconnected. Formally, spatial autocorrelation is defined in this model as the inverse in the sum of the differences in the environmental conditions, between each pair of connected communities. There are three arrangements of environmental conditions: lowest, intermediate and highest, such that the lowest arrangement have high environmental variation between the connected community pair and the highest have low environmental variation between the connected community pairs, that is communities closest to each other would expect to have similar environmental conditions. In terms of immigration around the metacommunity, it is defined in terms of the rate at which species came from the regional pool to each community. Note, that ref. ^66^ found that immigration does not change the overall patterns found, it just changes the minimum diversity levels.

Following ref. ^66^, we used a metacommunity of 100 communities on a grid network, seeded with a total number of species equal to 127 following ref. ^40^. Because this is a mechanistic model that uses normalized parameters, it is not possible to parametrize it using statistical data extracted from our populations. We simulated 10,000 alpha diversities with the parameters randomly selected from the following ranges. We set a small immigration rate *n* = 0–0.1 to indicate the inevitable lack of full sampling that is inherent within the fossil record (compare *n* = 0 in ref. ^66^). Dispersal (*ω*) ranged between 0.01 (highly dispersal limited) and 2 (wide dispersal), niche overlap (*h*) between 0.01 (high niche overlap) and 10 (low niche overlap). Spatial autocorrelation, used to indicate the impact of habitat heterogeneities on the taxa, followed ref. ^66^ where low spatial autocorrelation corresponded to a random arrangement of the differences in environmental conditions, high spatial autocorrelation minimized the differences in environmental conditions and intermediate autocorrelation corresponded to the midpoint between the low and high spatial autocorrelations. We set the observed values of alpha diversity for the Avalon, White Sea and Nama assemblages as described previously^23^ with *α*_Avalon_ = 20; *α*_White Sea_ = 36; *α*_Nama_ = 28. For each of these observed values, we performed ABC to estimate the posterior distributions for dispersal, niche overlap, immigration and spatial autocorrelation, using neutral networks with a tolerance of 0.05 (that is using the best 500 fitting models based on the distance between simulated and observed alpha diversity).

### Reporting summary

Further information on research design is available in the Nature Portfolio Reporting Summary linked to this article.

## Supplementary information

Reporting Summary

Peer Review File

## Figures and Tables

**Extended Data Table 1:**
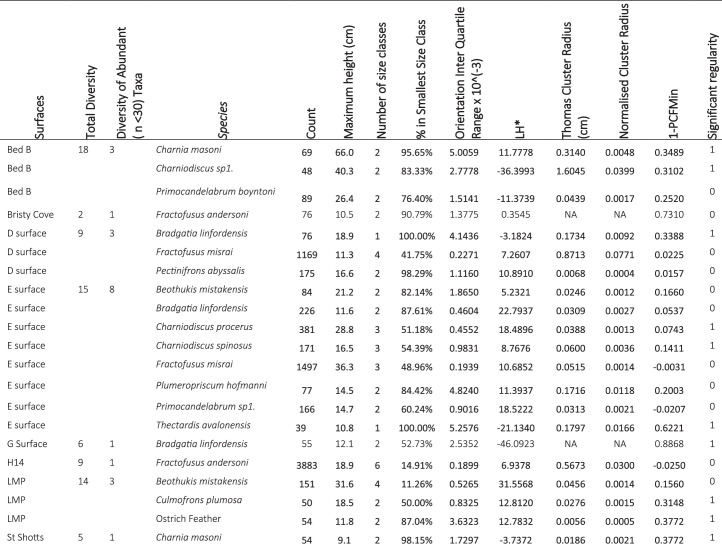
The input parameters for the Mann-Whitney tests and linear regressions

LH* is a measure of the patchiness/heterogeneity in the data. Note that where populations did not exhibit aggregations, such as the G surface *Bradgatia* and Bristy Cove *Fractofusus*, fitting cluster models was not appropriate, so were excluded from these analyses.

**Extended Data Table 2:**
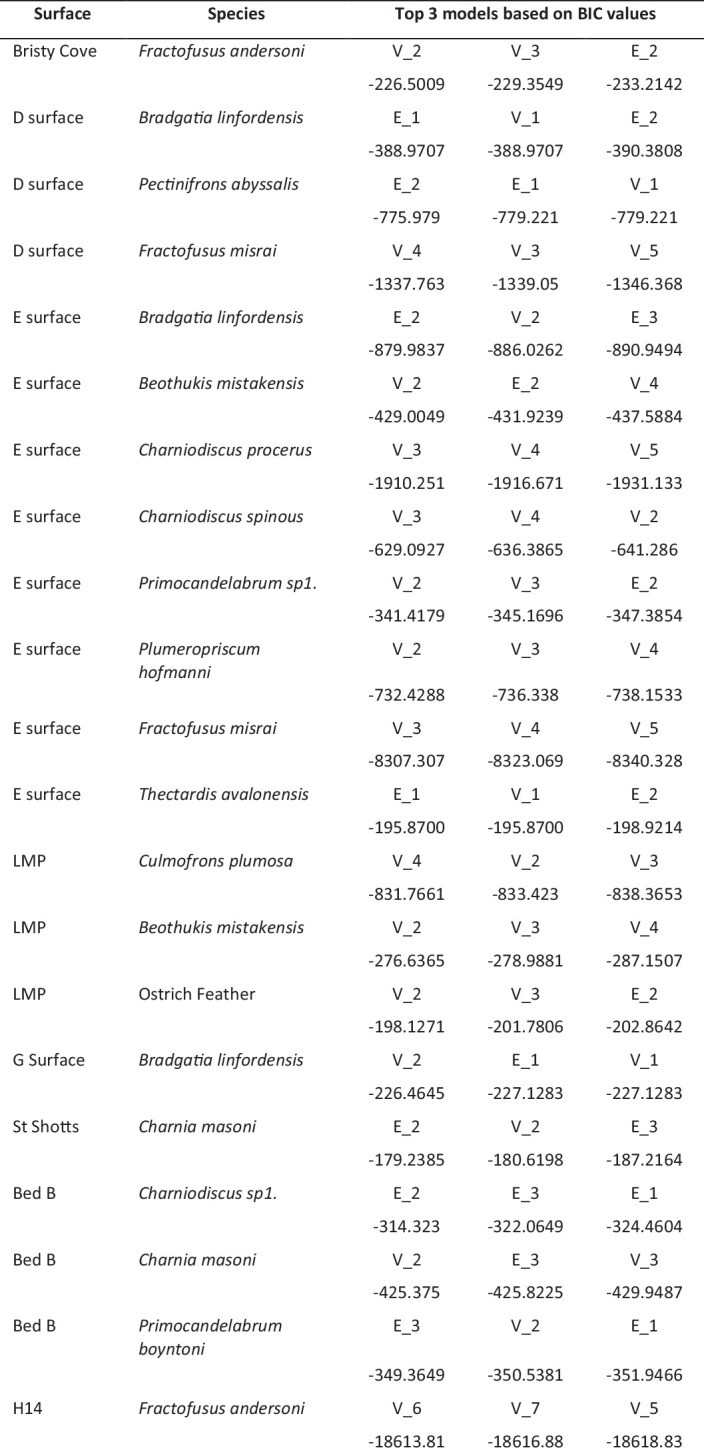
BIC values for the top 3 models found for the height distributions of the different species populations

V refers to models where the standard deviation of the best-fit model for each cohort within the population could vary, and E where the best-fit model have to have the same standard deviation. The numbers after the E or V refer to the number of cohorts in the best fit model. Note that not all the best fit models are significantly different from the second-best fit model.

**Extended Data Table 3:**
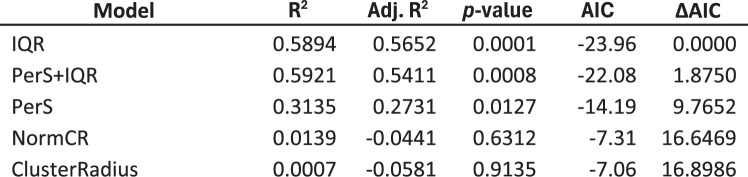
Summary of the stepwise regression for the variables (Extended Data Table 1)

variables (Extended Data Table 1) showing NormCR – Normalised cluster radius; DIQR – directionality inter quartile range; PerS – percentage in the smallest size class and LH* – lateral heterogeneity.

**Extended Data Table 4:**
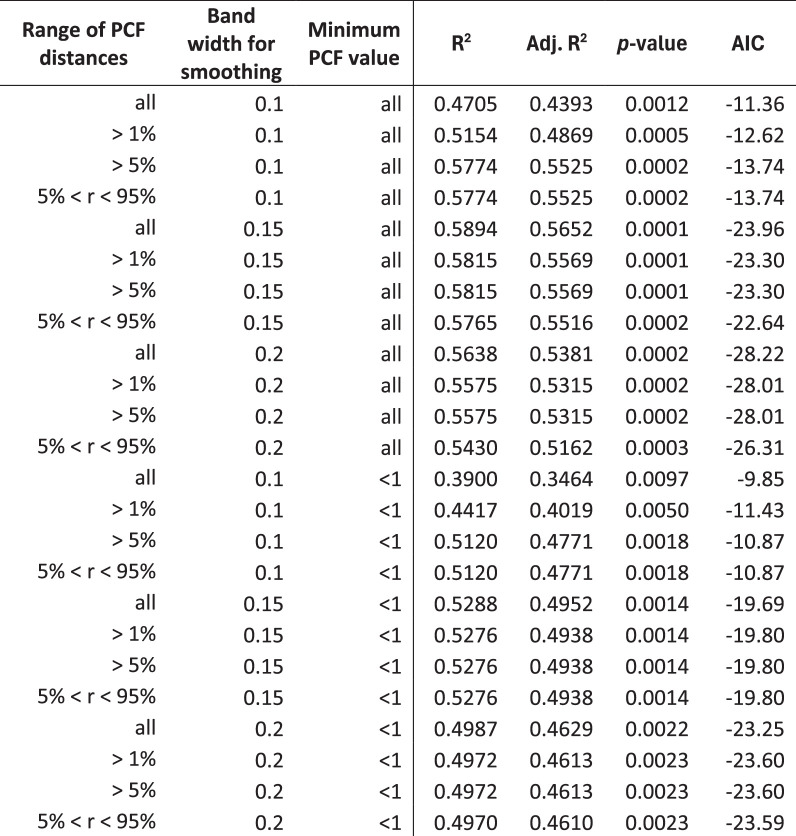
Summary of the sensitivity analyses for the regressions for the models

The model of 1-MinPCF ~ DIQR + PerS + NormCR. (Extended Data Table 1) showing NormCR – Normalised cluster radius; DIQR – directionality inter quartile range; PerS – percentage in the smallest size class.

**Extended Data Table 5:**
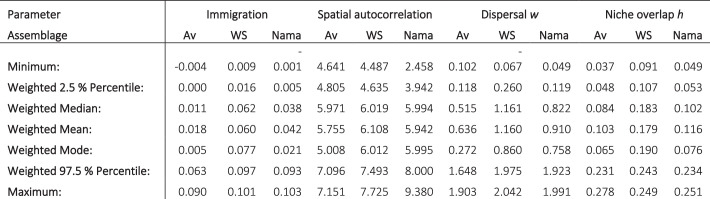
The summary statistics of the four estimated parameters of the mechanistic models

## Data Availability

Data for the fossil surfaces, the modern hydroids and coral and sponge community are available via GitHub at https://github.com/egmitchell/MitchellManica2026. Owing to geoconservation concerns, data from bed B cannot be made publicly available, with the full set available to researchers on request from the corresponding author.

## Code availability

Code is available via GitHub at https://github.com/egmitchell/MitchellManica2026.
