# Supplementary material for: The influence of reproductive mode on resource competition and diversity patterns in Ediacaran early animal communities
Source: Nat Ecol Evol. 2026 Jun 9;10(7):1234–44. doi: 10.1038/s41559-026-03094-2 (PMC13345964; doi:10.1038/s41559-026-03094-2)
Supplement: Supplementary file 2 — Peer Review File [file 41559_2026_3094_MOESM2_ESM.pdf]

nature portfolio

Peer Review File

The influence of reproductive mode on resource competition and diversity patterns in Ediacaran early animal communities

Corresponding Author: Dr Emily Mitchell

Version 0:

Decision Letter:

13th June 2025

*Please ensure you delete the link to your author homepage in this e-mail if you wish to forward it to your co-authors.

Dear Emily,

Your Article, "The influence of reproductive mode on resource competition and diversity patterns in Ediacaran early animal communities" has now been seen by three reviewers. The reviewers are clearly interested and intrigued by the paper, but reviewers 1 and 3 have some fairly serious concerns about the strength of the evidence base and the application of the methods. As they have some suggestions for how these might be improved, we would be willing to consider a revised version of the manuscript that addresses their concerns, but please bear in mind that we will be reluctant to approach the reviewers again in the absence of major revisions.

If you choose to revise your manuscript taking into account all reviewer and editor comments, please highlight all changes in the manuscript text file [OPTIONAL: in Microsoft Word format].

We are committed to providing a fair and constructive peer-review process. Please do not hesitate to contact us if there are specific requests from the reviewers that you believe are technically impossible or unlikely to yield a meaningful outcome.

If revising your manuscript:

* Include a “Response to reviewers” document detailing, point-by-point, how you addressed each referee comment. If no action was taken to address a point, you must provide a compelling argument. This response will be sent back to the referees along with the revised manuscript.

* If you have not done so already we suggest that you begin to revise your manuscript so that it conforms to our Article format instructions at http://www.nature.com/natecolevol/info/final-submission. Refer also to any guidelines provided in this letter.

* Include a revised version of any required reporting checklist. It will be available to referees (and, potentially, statisticians) to aid in their evaluation if the manuscript goes back for peer review. A revised checklist is essential for re-review of the paper.

Please use the link below to submit a revised paper:

Link Redacted

Note: This URL links to your confidential home page and associated information about manuscripts you may have submitted, or that you are reviewing for us. If you wish to forward this email to co-authors, please delete the link to your homepage.

If you wish to submit a suitably revised manuscript we would hope to receive it within 6 months. If you cannot send it within this time, please let us know. We will be happy to consider your revision so long as nothing similar has been accepted for publication at Nature Ecology & Evolution or published elsewhere.

Nature Ecology & Evolution is committed to improving transparency in authorship. As part of our efforts in this direction, we are now requesting that all authors identified as ‘corresponding author’ on published papers create and link their Open Researcher and Contributor Identifier (ORCID) with their account on the Manuscript Tracking System (MTS), prior to acceptance. This applies to primary research papers only. ORCID helps the scientific community achieve unambiguous

attribution of all scholarly contributions. You can create and link your ORCID from the home page of the MTS by clicking on 'Modify my Springer Nature account'. For more information please visit please visit www.springernature.com/orcid.

Please do not hesitate to contact me if you have any questions or would like to discuss the required revisions further.

Thank you for the opportunity to review your work.

[redacted]

Reviewer expertise:

Reviewer #1: palaeobiology, community ecology

Reviewer #2: palaeobiology, community ecology

Reviewer #3: spatial ecology and spatial point pattern analysis

Reviewers' comments:

Reviewer #1 (Remarks to the Author):

This paper analyses the spatial distributions of taxa from the famous Ediacaran-aged Mistaken Point fossil surfaces, with a specific eye towards identifying ecological signatures associated with putative stolon-type structures (which might be linking individuals of some species in the sediment subsurface). In brief, the presence of stolons linking individuals brings with them predictions surrounding ecology and distributions on the bedding plane. Members of individual colonies should show limited evidence for intra-specific competition ('heteromyopia'), and – given that the growth of new individuals is determined by benthic stolon growth (rather than via settling, and thus influenced by the ambient current directions) – should form more circular distributions on the seafloor. The authors find a link between more circular colonies and evidence for competition at different spatial scales, and thus evidence for heteromyopia in several of the studied surfaces. In other words, they find individuals of different species clustering closer together than individuals of the same species…suggesting inter-specific competition at extreme local scales. Using this, the authors suggest that the waxing and waning of stoloniferous reproduction among early metazoan groups through the Ediacaran may be responsible for the observed changes in diversity ~575-538 Ma; a metacommunity model designed to simulate the effects of dispersal and immigration on local species richness patterns provides an approximate match with alpha diversity trends over the interval. The authors conclude that stoloniferous reproduction – more specifically its loss – likely played an important role in the diversification of Ediacaran macrofauna.

This is a neat paper that pioneers some sophisticated methods, and which that presents an interesting idea. However, parts of the paper are quite opaque…and I think the text would benefit from either some clearer explanations, or some figures that illustrate (either actually, or schematically) the patterns the authors are identifying. Moreover, I have some questions and criticisms surrounding the more 'mechanistic' parts of the paper, which may benefit from some re-thinking and/or expanded discussion.

Stolons. – I am excited by the stolon model for rangeomorph paleobiology, but I think the best evidence for them is still inferential (rather than physical), and so the paper should acknowledge this ambiguity. For example, the statement, "Stoloniferous reproduction has been directly observed in five Avalonian taxa…" (line 53) is too strong; the evidence based in the Liu and Dunn 2020 paper is equivocal (most filaments on the Mistaken Point surface don't seem to really attach to anything at all), and the Dunn et al. 2025 paper doesn't figure any clear stolons…certainly not anything extending more than a cm beyond the margin of the fossils. Added to this, Becker-Kerber et al. 2021 presented some compelling evidence to suggest that many of the filamentous structures on the Mistaken Point surface may be sulfur-oxidising bacteria. I don't think acknowledging this ambiguity weakens the current paper (I actually think there's better evidence for stoloniferous reproduction in these patterns, than in any of the studies claiming to have identified them)…and would give a better representation of the current state of knowledge.

Isotropic/anisotropic clusters. – Without any illustrations, or images of the fossil surface, I'm struggling to envisage what these clusters look like on the bedding plane, or how they might overlap in a way that would produce closer spacing of competing species, than individuals of the same species. Part of this is confused by some of the wording early in the main text; for example, it's stated (line 73) that, "Attachment via stolons results in isotropic clusters…i.e., are circular rather than ovate", but also on that, "F. andersoni shows the lowest levels of isotropy…consistent with previous work that inferred stoloniferous reproduction" (line 117). There may be a typo somewhere in here, but - reading it – it made me feel as though I wasn't following the logic structure. I think a lot of this could be cleared up using a figure or suitable illustration – for example, is there any reason why the authors can't show an image of a bedding plane, with clusters of different species highlighted to give the reader an idea for the scale and overlapping distribution of clusters belonging to different species? Alternatively, this could be done via a schematic diagram…but, either way, I think some sort of visualization will be necessary for most readers to get their heads around the complex spatial relationships being described here. Similarly, I found the boxplots shown in Figure 1 quite confusing. I get that this is designed to show a separation between species with/without inferential evidence

for stolons, and a relationship with the shape of clusters, but the wording in the caption apparently defining clusters ('interquartile range of point to point orientations') isn't used elsewhere, and so is a bit opaque. The wording in the text, ("We…quantify cluster directionality by measuring the interquartile range of the orientations of all specimens relative to each other…") makes is sound as though clusters are defined by the orientation of individual fossils, whereas elsewhere it more sounds as though isotropy is being used to describe the outline of the community on the bedding plane (something that would make more sense in context of the fluid dynamics argument invoked on line 76). In sum, I think the methods behind the identification and measurement of clusters needs much better explanation.

Figure 2. – I also found this figure slightly confusing; the logic in the caption is well explained (if members of two different species are spaced closer together than individuals belonging to either of the species themselves, then this suggests inter-specific competition at smaller scales than intra-specific competition), but there are some slight mismatches with the text. For example, the text states, "the largest heteromyopic difference…was with Fractofusus and Charniodiscus procerus…where the inter-specific segregation occurred 1.21m before the intra-specific segregation of Charniodiscus procerus, and 2.85m than Fractofusus" (line 152)…however…looking at the plot, I can't make these measurements match up with what's shown on that line. It would perhaps help if the authors could highlight species pairs in the y-axis where heteromyopia is detected, and perhaps arrow specific examples that are highlighted in the text.

Links to the 'second wave' of Ediacaran reproduction. – I'm a bit skeptical of the broader model being outlined here. On a specific level, the notion that evolving, and motile bilaterians may have broken stoloniferous connections in the subsurface is highly speculative, and is at odds with what we know about White Sea-aged environments and tracemakers. For a start, the microbial mat surfaces that characterize the White Sea (termed 'textured organic surfaces' by Tarhan and colleagues) are suggestive of thicker, more robust, and more complex mat communities than anything seen in the Avalon, and clearly presented a substantial barrier to both vertical and horizonal movement…what few ichnotaxa we have from this interval are either extremely small, or are limited to the top few mm of the mat surface (or conceivably, between shallow sediment laminae and the mat itself). Moreover, to the best of my knowledge nothing resembling a stolon has been identified from any surfaces beyond the Avalon, despite several (at least potentially) stoloniferous groups persisting into this interval. At a broader level, there's perhaps a hint of a circular argument being developed here…from the way the text is written, it makes it sound as though the breakage of stoloniferous connections by newly-evolving and motile metazoans, may have led to increased competition and thus the evolution of motile metazoans. Modes of reproduction cannot then be the only implicit driver - more complex and motile metazoans must thus have evolved first, for increasing competition as a driver of of evolutionary novelty to make sense. I don't mean to say that this model is wrong – it's an innovative idea and deserves to be out there – but, as currently framed, it doesn't come across as internally consistent, and doesn't match with any observations we have from the Avalon-White Sea transition.

Reviewer #2 (Remarks to the Author):

I really loved reading this work by Mitchell and Manica. This manuscript is quite an interesting and well-structured paper that proposes an ecological mechanism— heteromyopia driven by stoloniferous reproduction— to explain key macroevolutionary patterns in early animal diversification during the Ediacaran period.
The work presents a novel and plausible hypothesis, supported by quantitative spatial analyses of Ediacaran fossil assemblages and mechanistic modeling. It tackles a significant question: what controlled the tempo and mode of early animal diversification? The authors argue convincingly that the prevalent mode of reproduction (stoloniferous asexual reproduction) in early Ediacaran (Avalonian) communities led to a peculiar competitive dynamic called heteromyopia, which in turn limited selection pressures and constrained diversification until a shift towards sexual reproduction and/or increased mobility occurred, heralding the "Second Wave" of Ediacaran diversification.
Although there are assumptions about certain aspects of reproductive mode and other ecological factors at play, I think the arguments put forward in this manuscript are convincing and am happy to recommend this paper be accepted with some very minor changes and suggested edits, which are more like copyediting issues (noted below). I would like to congratulate the authors on writing such a compelling manuscript.

Potential Copyediting Errors & Minor Issues:
• Page 2, Line 41: "in the Avalon there are instances" -> maybe to make clearer "in Avalonian communities there are instances" or "within Avalon assemblages there are instances" for consistency with other phrasing.
• Page 4, Line 74: "that is clusters which do not have any directionality, i.e. are circular" -> "that is, clusters that do not have any directionality (i.e., are circular..." - Minor punctuation.
• Page 5, Line 114: very minor: "segregation up until (or over) the point where the observed data crosses PCF=138." - "up to" is sometimes preferred over "up until."
• Page 6, Line 121: "In contrast, the Bristy Cove Fractofusus showed high levels of directionality, likely due to the relative immaturity of the community (as inferred by the small body sizes and large percentage in the smallest size class, Table 1), suggesting that the population had not enough time to reproduce." -> "...suggesting that the population had not had enough time to reproduce" or "...suggesting the population lacked sufficient time to reproduce."
• Page 8, Line 163: "Heteromyopic relationships were present if the interspecific estimate (purple point) was found at smaller minimum distance than either of the intra-specific once (blue/red)." -> "...than either of the intra-specific ones (blue/red)."
• Page 10, Line 191: "smaller spatial scales than intra-specific competition for one taxon indicating" -> "smaller spatial scales than intra-specific competition for one taxon, indicating..." (comma for clarity).
• Page 12, Line 252: "increase in Ediacaran diversity around 550 Ma7. has the potential be explained" -> "...has the potential to be explained..."

• Page 13, Line 277: "7.924 Thus, our mechanistic model..." - The "7,924" seems like a stray citation number or error. Did you mean 7, 9 and 24?
• Source 213, Page 23, Line 481 (Figure 2 Caption): "Minimum distance of segregation" - "minimum" should not be capitalized unless it's part of a formal name.
• Page 24, Line 504: "where isotropy was indicated by only small variations" -> "where isotropy is indicated by only small variations" (present tense for describing a general method/interpretation).
• Page 25, Line 532: "For all populations--(,)-- the number of normally distributed cohorts within the populations were found 64" -> "...within the populations was found".
• Page 26, Line 558: "Thomas clusters are associated with reproductive processes 14,32,38,66 so a Thomas Cluster sizes are good way" -> "...so Thomas Cluster sizes are a good way..."
• Page 27, Line 595: "αavalon=20; αwhitesesa=36; αnama=28" -> "whitesesa" is meant to be "White Sea"
• Page 29, Line 605: "Note that some cases, such as the G surface Bradgatia where Thomas Clusters are not a good model fit, the cluster size tends to zero." -> "...Bradgatia, where Thomas Clusters are not a good model fit, the cluster size tends towards zero." (please note the use of commas elsewhere as well)

Reviewer #3 (Remarks to the Author):

This manuscript uses data on the spatial distribution of in-situ, sessile benthic organisms that have been preserved as fossils to derive hypotheses to explain the burst of rapid diversification of the Ediacaran animal communities after a longer period of little change. The idea is that widespread reproduction via stolon in the first animals led to dispersal limitation of the dominant species and coexistence of weaker competitors operating under reduced selection pressure. The hypothesis is that disappearance of the stoloniferous reproductive strategy should increasing competition and selection pressure and lead to the observed burst of diversification (the Second Wave). The hypotheses derived from the reanalysis of mapped fossil communities are then tested by simulations of a metacommunity model.

I find the possibility to obtain and analyse maps of organisms some 500 million years ago fascinating, and the idea to explain the burst of diversification of the Second Wave by changes in the spatial structure of the communities (driven by changes in the reproductive strategy) very interesting. However, my impression is that the evidence presented in favour of the hypotheses is not very strong. Parts of the spatial analysis (the determination of the scales of segregation) are a bit weak and the mechanistic model is not very convincing since it seems not to match the scales and the biology of the system (and the data extracted from the fossil maps) very well. A specifically tailored spatial model that can generate spatial data that can be directly compared to that extracted from the fossils (and includes stolon reproduction vs. water-borne reproduction) would be needed to provide more conclusive evidence for the interesting hypothesis.

lines 18-21, 24-28
This is an interesting hypothesis

lines 75-79
Perhaps you could show two contrasting spatial maps (circular vs. elongated clusters) of the organisms to illustrate the dispersal clusters?

lines 82-95
This is an interesting analysis.

line 88
What is the first and second factor?

lines 106-114, 140-156
Looking at the graphs of Extended data Fig. 2 it looks that you have some issues with too small bandwidths, which lead to somewhat rugged pair correlation functions. This makes your objective to determine scales of segregation difficult to attain since some of the fluctuations of the function may be due to stochastic noise.

Given that competition is a more cumulative mechanisms, you may use instead a variant of the K-functon(K(r)/pi r^2), which is the direct cumulative analogue to the pair correlation function: it gives the mean (normalized) neighbourhood density of points within distance r of the focal individuals). This version of the K-function has no issue with the bandwidth and will produce smoother estimates.

Given that the width of the simulation envelopes depends very much on the number of individuals of the patterns (it is basically proportionally to 1/N), you may also consider to just look at the distances where the empirical summary functions has values below 1 and use the lowest value within a competitive neighbourhood as measure of strength of competition.

Line 170-179
I am curious to see what you get out using a more stable summary function to estimate segregation.

Figure 3
The minimal value of the pair correlation function segregation is zero, which indicates maximum segregation (i.e., no neighbour at the given distance). Therefore, the y-axis with values < 0 does not make sense. The range of values in the

regression analysis must be constrained to >=0.

line 264
It is unclear how the mechanistic model incorporates heteromyopia at the scales extracted from the spatial analysis.

lines 273-277
If the model captures the mechanisms occurring at the three assemblages…

Figure 4
Although the Avalon posterior distribution has a peak at low dispersal parameters, all three distributions are quite flat. The dispersal parameter need to be explained in the main text.

line 445-446
There is something wrong with the reference.

line 504
Fig. 1?

lines 531-535
Unclear what you did and why you did it.

lines 547-552
How did you correct for the non-rectangular shape of the observation area?
What is the pd value?
See also my comments about the rugged pair correlation functions above.

linen556
You should briefly explain the Thomas process and how it can be used to determine the cluster size.

lines 576-599
The model should be explained in more detail. Looking at ref. 54 it is unclear how this model fits your assumptions/hypotheses. You base your hypotheses on neighbourhood analyses of individual organisms (and stolon reproduction), whereas the model is a metacommunity model that looks at patches (of local communities), connected by dispersal. Your data represent maps of individuals within a certain area, which may correspond to single patches of the metacommunity model. Additionally, your hypothesized distance of dispersal are rather inside the patch than between patches as assumed in the metacommunity model.

Version 1:

Decision Letter:

10th March 2026

Dear Dr. Mitchell,

Thank you for submitting your revised manuscript "The influence of reproductive mode on resource competition and diversity patterns in Ediacaran early animal communities" (NATECOLEVOL-25041310A). It has now been seen again by the original reviewers and their comments are below. The reviewers find that the paper has improved in revision, and therefore we'll be happy in principle to publish it in Nature Ecology & Evolution, pending minor revisions to satisfy the reviewers' final requests and to comply with our editorial and formatting guidelines.

If the current version of your manuscript is in a PDF format, please email us a copy of the file in an editable format (Microsoft Word or LaTex)-- we can not proceed with PDFs at this stage.

If you have not done so already, please ensure that you also email us a completed copy of the Reporting summary :

Reporting summary: https://www.nature.com/documents/nr-reporting-summary.pdf

We are now performing detailed checks on your paper and will send you a checklist detailing our editorial and formatting requirements in about a week. Please do not upload the final materials and make any revisions until you receive this additional information from us.

Thank you again for your interest in Nature Ecology & Evolution. Please do not hesitate to contact me if you have any questions.

[redacted]

Reviewer #1 (Remarks to the Author):

The authors have done a nice job with this revision – I find the text much easier to follow, the new figures do a great job of illustrating the patterns in an intuitive way, and any lingering circularity has been removed. Barring a few, tiny typos and formatting errors, I think that this is now ready for publication, and I look forward to seeing it in print. These last few things are numbered below:

1. “Importantly, heteromyopic dynamics are hard to distinguish from neutral dynamics based on most ecological metrics, such as species area distributions [23]” (line 44); I believe the Darroch et al. paper analyzed species-abundance distributions, rather than species-area distributions.
2. Line 200; double comma.
3. Line 222; ditto.
4. Line 774; ditto.
5. “As such, our data is a good reflection of documented early animal life” (line 246); this same sentence is given in the first line of this section, and so I think here it’s redundant.

Reviewer #2 (Remarks to the Author):

According to my reading of the new version of the manuscript in the context of the comments by other reviewers and mine, the authors have successfully addressed the conceptual and methodological critiques raised during the initial review phase. I had initially overlooked the mobility problem raised by reviewer 1, and I appreciate the authors’ trying to resolve it using an environmental stability argument. The original manuscript relied on a seemingly circular argument where motile metazoans were both the cause and the result of increased competition through stolon breakage. In response, the authors have now reframed the macroevolutionary trigger as a transition driven by environmental stability. This new framing is more biologically parsimonious and better reflects the geological record of these environments.
Methodologically, the authors also seem to provide a good response to Reviewer 3’s concerns regarding the ruggedness of the Pair Correlation Functions (PCF) and potential stochastic noise. Rather than switching to the suggested K-function, which they argued would mask scale-specific segregation, the authors performed an exhaustive sensitivity analysis across different permutations of kernel bandwidths and distance ranges.
Furthermore, the addition of new figures, specifically Figure 1 for visualizing heteromyopia and Figure 2 for interpreting isotropic rose diagrams, directly resolves the opacity issues noted by all reviewers, providing a clear visual bridge between complex spatial point patterns and biological interpretations.
I am happy with this round of revisions and recommend the paper for acceptance.

Reviewer #3 (Remarks to the Author):

The authors responded well to my comments. The new figures are a great addition, and I am pleased with the sensitivity analysis, which increases confidence in the robustness of the approach. Overall, the manuscript now presents an intriguing hypothesis about changes in life many millions of years ago and provides solid evidence in its favour. This makes it really special.

However, on the downside, by closely re-reading the manuscript I found several points where the authors could improve readability. For example, I would prefer the more traditional way of writing figure captions, where each panel is explained in turn (and each panel should be labelled and referred to in the text!). Also, technical terms are often inconsistent and confusing, with many different terms being used for one quantity. Additionally, some figures are of poor graphical quality and must be improved. Finally, paragraphs are often too long and should be split. All of these issues could be resolved with little effort

Line 41
Perhaps start a new paragraph here to guide the attention of the reader to the phenomenon of heteromyopia. In general, your paragraphs tend to be too long, please check throughout the text.

Figure 1
Figure 1 is a good idea, but panels a) and b) are of poor graphical quality due to the coarse dashed lines, and the arrows in panel c) are unclear. Perhaps the peak of the arrows should be placed at the centre of the corresponding black specimens. In panels a) and b), the dots should be larger, and all the circles should be solid.

line 84
I would reduce the scope of this section to focus solely on the identification and quantification of stoloniferous reproduction, and open another section for the identification and quantification of resource competition

Figure 2

The new figure 2 is nice, you could also provide the values of interquartile range for each isotropy plot. Additionally I would recommend the traditional way of a figure legend, explaining each panel a), b, … i) individually in turn. You still can simplify e.g., e) same as d, but for the Thomas cluster process with a 2x directionality shown in a, f), ….

Generally, to help the reader, please label all panel with a, b, c, … and refer then in the text to both the figure number and the associated panel label.

line 106-113
It is unclear what your measure is. In general, the use of technical terms is often inconsistent. It would be very helpful for the reader if you could define a term unambiguously and then use it consistently for a given quantity. For example, you use 'rose diagram' versus 'isotropy plot', 'relative isotropy' versus 'isotropy' versus 'interquartile range' versus 'IQR', and so on.

Here you use all the terms “a metric to quantify cluster directionality”, “relative isotropy”, “interquartile range”, “IQR”, etc. to refer to the same thing. So, for example, this paragraph could read:
We used the interquartile range (IQR) of all point-to-point orientations within a population to quantify cluster directionality. The IQR was visualised by plotting the point-to-point orientation measurements as rose diagrams, where small values indicate isotropy (i.e. round isotropy plots; Fig. 2b, d and g) and high values indicate anisotropy (i.e. ovate isotropy plots; Fig. 2e, f, h and i).

Line 113
I would start here a new paragraph, since you jump to a new issue (the 3 factors).

line 126
I would open here a new section “Identification and quantification of resource competition”. This would make it easier to navigate through your manuscript.

lines 128 – 145
Using the same terms for inter-specific and intra-specific measures is confusing. I would suggest making some small changes to your terminology to help readers recognise which analysis you are referring to. For intra-specific measures, you could use the established terms “spatial aggregation” (or simply “aggregation”) and “regularity”, and for inter-specific measures, you could use the terms “positive association” (or “attraction”) and “segregation”.

line 137-145
You have to mention that you use the univariate pair correlation function for intra-specific analysis and the bivariate PCF for inter-specific analysis. In an intra-specific analysis, values below one corresponding to spatial regularity, above 1 to spatial aggregation. In an inter-specific analysis, values below one corresponding to spatial segregation, above 1 to spatial association (or attraction).

Line 147
We started by applying our new directionality measure IQR to 21 (…)

148
What is “IQR”, this is not previously defined in the text, but only in the figure 3 caption.

line 171
“which would indicated effects of habitat associations” since you talk about an intra-specific analysis. Habitat segregation makes sense for two different species.

lines 173, 174, 176, 197, 200, 208, etc
It is “regularity”, not “segregation”

line 177
Start a new paragraph

Figure 4
The idea of the figure is nice, but the figure design must be improved. The labels with the species names are far too small, and the coarse dashed lines should be solid lines with different colour to distinguish the different analyses. Each of the 5 cases should have an own label.

line 196
Introduce a new section “Relationship between stoloniferous reproduction and strength of intra-specific regularity”

line 200
It should be Fig 5 I guess.

lines 207-212, 137
Ok, use of the pair correlation functions is fine. You showed that the results are insensitive to the bandwidth (and distance range), which is important indication of robustness.

line 209
Extended Data Table 4 I guess?

Figure 5
The new Figure 5 is also a nice addition! It is a good idea to brake it down in terms of predictor variables.

line 262
Introduce a new paragraph

lines 272-297
Too long paragraph, split

line 298
Add a new section e.g., “A mechanistic model to investigate the effect of dispersal limitation” to help navigation through your paper.

line 306
Introduce a new paragraph to allow the reader to quickly find the model description.

line 306-317
Ok, it is fine that the mechanistic model includes heteromyopia only indirectly via dispersal limitation. However, you should state explicitly in the text that heteromyopia is in the model a consequence of high dispersal limitation, so that the reader can re-connect with the arguments in the summary and the introduction, as well with the results of the spatial analysis.

lines 673
I am also happy with the additional explanation of the edge correction method and the Thomas cluster methods. But start with a new paragraph.

line 719
Add “describing 100 communities on a grid network.”

Line 739
The description of spatial autocorrelation is confusing. Maybe just say that high autocorrelation means that communities closes to each other have similar environmental conditions, whereas low autocorrelation means that the environmental conditions of nearby communities are uncorrelated.

Open Access This Peer Review File is licensed under a Creative Commons Attribution 4.0 International License, which permits use, sharing, adaptation, distribution and reproduction in any medium or format, as long as you give appropriate credit to the original author(s) and the source, provide a link to the Creative Commons license, and indicate if changes were made.
In cases where reviewers are anonymous, credit should be given to 'Anonymous Referee' and the source.
The images or other third party material in this Peer Review File are included in the article’s Creative Commons license, unless indicated otherwise in a credit line to the material. If material is not included in the article’s Creative Commons

license and your intended use is not permitted by statutory regulation or exceeds the permitted use, you will need to obtain permission directly from the copyright holder.
To view a copy of this license, visit https://creativecommons.org/licenses/by/4.0/

Reviewer #1 (Remarks to the Author):

This paper analyses the spatial distributions of taxa from the famous Ediacaran-aged Mistaken Point fossil surfaces, with a specific eye towards identifying ecological signatures associated with putative stolon-type structures (which might be linking individuals of some species in the sediment subsurface). In brief, the presence of stolons linking individuals brings with them predictions surrounding ecology and distributions on the bedding plane. Members of individual colonies should show limited evidence for intra-specific competition ('heteromyopia'), and – given that the growth of new individuals is determined by benthic stolon growth (rather than via settling, and thus influenced by the ambient current directions) – should form more circular distributions on the seafloor. The authors find a link between more circular colonies and evidence for competition at different spatial scales, and thus evidence for heteromyopia in several of the studied surfaces. In other words, they find individuals of different species clustering closer together than individuals of the same species…suggesting inter-specific competition at extreme local scales. Using this, the authors suggest that the waxing and waning of stoloniferous reproduction among early metazoan groups through the Ediacaran may be responsible for the observed changes in diversity ~575-538 Ma; a metacommunity model designed to simulate the effects of dispersal and immigration on local species richness patterns provides an approximate match with alpha diversity trends over the interval. The authors conclude that stoloniferous reproduction – more specifically its loss – likely played an important role in the diversification of Ediacaran macrofauna.

This is a neat paper that pioneers some sophisticated methods, and which that presents an interesting idea. However, parts of the paper are quite opaque…and I think the text would benefit from either some clearer explanations, or some figures that illustrate (either actually, or schematically) the patterns the authors are identifying. Moreover, I have some questions and criticisms surrounding the more 'mechanistic' parts of the paper, which may benefit from some re-thinking and/or expanded discussion.

Thank you for your kind words about our paper. As described in detail below we have created two new figures that show heteromyopia and isotropic clusters. Further to your comments on our discussion we have re-thought it, and agreed there was some issues and circularity, so have reframed in terms of environmental triggers of sexual reproduction.

"Stolons. – I am excited by the stolon model for rangeomorph paleobiology, but I think the best evidence for them is still inferential (rather than physical), and so the paper should acknowledge this ambiguity. For example, the statement, "Stoloniferous reproduction has been directly observed in five Avalonian taxa…" (line 53) is too strong; the evidence based in the Liu and Dunn 2020 paper is equivocal (most filaments on the Mistaken Point surface don't seem to really attach to anything at all), and the Dunn et al. 2025 paper doesn't figure any clear stolons…certainly not anything extending more than a cm beyond the margin of the fossils. Added to this, Becker-Kerber et al. 2021 presented some compelling evidence to suggest that many of the filamentous structures on the Mistaken Point surface may be sulfur-oxidising bacteria. I don't think acknowledging this ambiguity weakens the current paper (I actually think there's better evidence for stoloniferous reproduction in these patterns, than in any of the studies claiming to have identified them)…and would give a better representation of the current state of knowledge."

We appreciate the discussions around the nature of the filaments so have modified our text on lines 65-70 (all line numbers refer to the tracked changes version):

> "Stoloniferous reproduction[24,25] within Avalonian communities was first inferred based on the size-structured spatial arrangement of specimens[16], with further evidence provided by numerical and computational fluid dynamics[26]. In terms of physically preserved stolon, filaments have been found connected to five Avalonian taxa[24,25], suggesting preserved stolon, although non-stoloniferous filaments are also likely present[27,28]."

We agree that there could be many different affinities for the filaments preserved in Avalonian assemblages, and currently have a student with Alex Liu working on identifying the different types and possible affinities of these filaments. In terms of the Becker-Kerber et al. 2021 filamentous structures, while these are undoubtedly very interesting, and could well be preserved on some Avalonian bedding planes (for example Pigeon Cove, Mistaken Point), the relatively densities, interwoven elements and dimensions are very different to the filaments that are preserved on the fossiliferous surfaces within this study, and those that attach to Avalonian body fossils, so the above phrasing is appropriate.

Isotropic/anisotropic clusters. – Without any illustrations, or images of the fossil surface, I'm struggling to envisage what these clusters look like on the bedding plane, or how they might overlap in a way that would produce closer spacing of competing species, than individuals of the same species.

We appreciate the difficulty of visualising these patterns, so have added two new figures. These patterns are not normally apparently with the naked eye when viewed in modern or fossil communities, so we have simulated some data to emphasize what is being seen. First, we added a new figure which showed simulated and extant observed isotropic and anisotropic patterns (Fig. 2), and gave more detail about how they should be understood on Lines 111-119:

> "We developed a metric to quantify cluster directionality by first measuring orientation between each point (here fossil specimen) to all the other points (fossil specimens), then defining the relative isotropy as the variability of these orientations, measured as the interquartile range (i.e. isotropy is measured by the interquartile range of all of the point-to-point orientations across the population). This directionality measure was visualised by plotting each of these point-to-point orientation measurements as a rose diagram, with isotropy indicated by small values, i.e. round isotropy plots, (Fig. 2 b,d,g), and anisotropy by high values, i.e. ovate isotropy plots (Fig. 2e,f,h,i)."

And on lines: 686-696:

> "We first simulated Thomas Cluster distributions (Fig. 2a), with no inherent directionality, which showed a relatively isotropic, namely circular rose diagram (Fig. 2d). We then applied a 2x transformation to these Thomas Cluster points along the x-axis only (Fig. 2b), which showed an ovate rose diagram (Fig. 2e), with a 3x transformation (Fig. 2c), showing a more elongated rose

plot (Fig. 2f). To illustrate how IQR varies with different reproductive modes, we ran our analyses on three observed examples. First, an extant hydroid stoloniferous colony[74] to demonstrate the high isotropy of stoloniferous reproduction (Fig. 2g), and then for the sponge *Advhena magnifica* in two areas of an extant deep-sea coral and sponge community[30] exhibiting high anisotropy, with the anisotropy varying due to different current direction on the two different areas (Fig. 2h,i)."

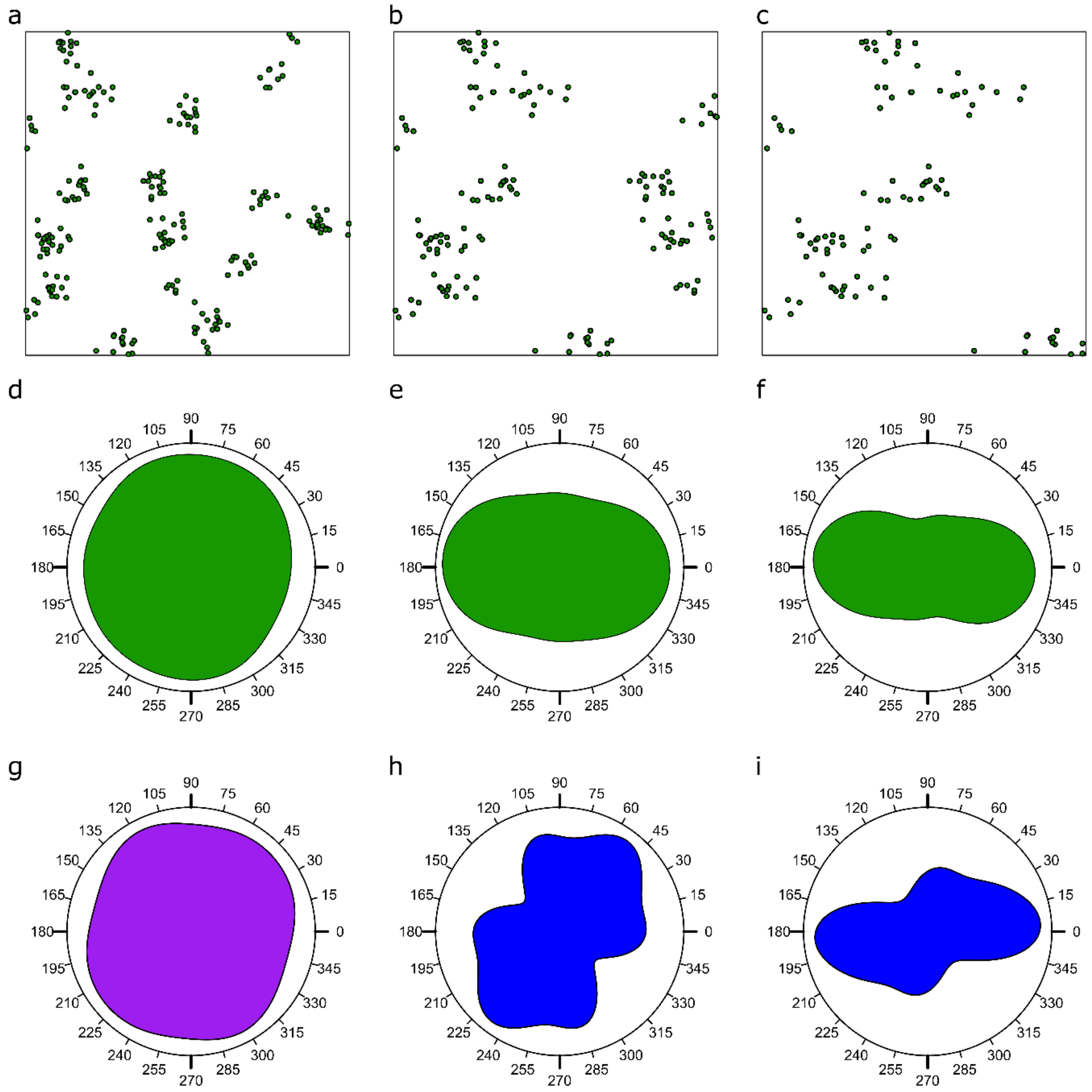

"Figure 2: Examples of different levels of isotropy. **a)** Simulated Thomas clusters with their associated isotropy plot (as one would expect for clonal organisms) **d). b)** Shows the same Thomas Clusters with a 2x directionality on the x-axis (as might be caused by broadcast spawning), shown by the elongated isotropy plot in **e)**. **c)** shows the simulated Thomas Clusters with a 3x directionality, and an associated more ovate isotropy plot in **f)**. **g)** observed examples of a hydroid colony connected by stolon, and for the deep-sea sponge *Advhena magnifica* in two sections of a deep sea coral and sponge community subject to different current directions in **h)** and **i)** (from Ref. 30)."

Secondly, in order to visualise heteromyopia, we have added a new Figure 1, lines 52-60 which describes how and why competition between species can occur on smaller spatial scales than for within species competition.

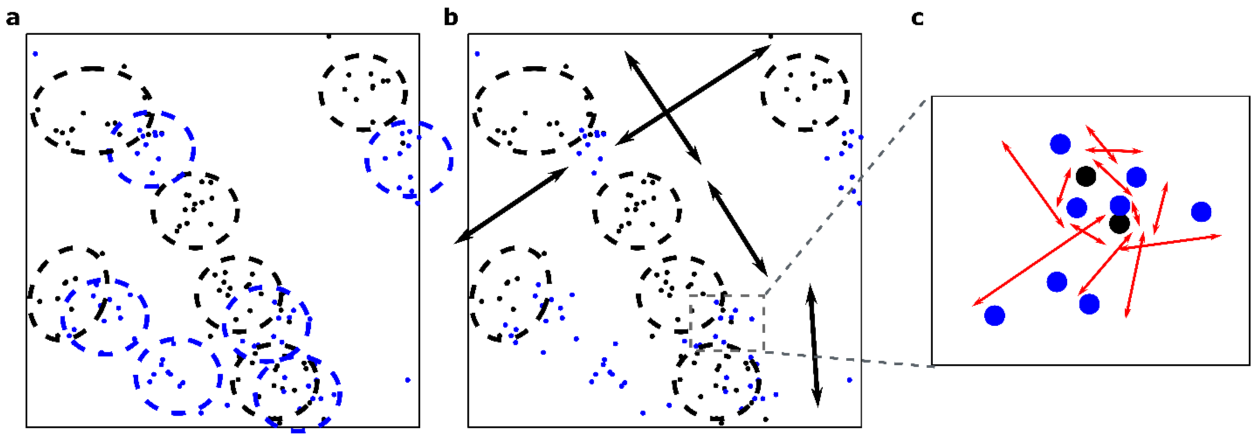

"Figure 1 Schematic diagram demonstrating heteromyopia. The blue and black dots represent different species. **a)** If both species are stoloniferous, then all specimens within a cluster (given by the dashed line), remained connected. In this instance, the spatial scales of competition would not be within the cluster (dashed circle), but only between them, shown by the black arrowed lines in **b)**. However, competition can still occur between two different species, for example within the grey dashed box in **b)**, which would occur on spatial scales smaller than within species where the two clusters overlap (as seen in **a**), and shown by the red arrows in **c)**, which depicts the blue specimens that the two black specimens would compete with."

Part of this is confused by some of the wording early in the main text; for example, it's stated (line 73) that, "Attachment via stolons results in isotropic clusters...i.e., are circular rather than ovate", but also on that, "F. andersoni shows the lowest levels of isotropy...consistent with previous work that inferred stoloniferous reproduction" (line 117). There may be a typo somewhere in here, but - reading it – it made me feel as though I wasn't following the logic structure.

Our apologies for this typo, which is now corrected on Line 155-158:

"H14 *Fractofusus andersoni* showing the highest levels of isotropy and *Thectardis* on E surface showing the lowest levels of isotropy ($IQR_{H14: \textit{Fractofusus}}$= 0.0002; $IQR_{E: \textit{Thectardis}}$= 0.0053; Fig 3; Extended Data Table 1)".

I think a lot of this could be cleared up using a figure or suitable illustration – for example, is there any reason why the authors can't show an image of a bedding plane, with clusters of different species highlighted to give the reader an idea for the scale and overlapping distribution of clusters belonging to different species? Alternatively, this could be done via a schematic diagram...but, either way, I think some sort of visualization will be necessary for most readers to get their heads around the complex spatial relationships being described here.

We agree that visualisation is needed, but the clusters aren't obvious from the photographs of the bedding planes, which is why we have produced a schematic diagram instead (Figure 1).

Similarly, I found the boxplots shown in Figure 1 quite confusing. I get that this is designed to show a separation between species with/without inferential evidence for stolons, and a relationship with the shape of clusters, but the wording in the caption apparently defining clusters ('interquartile range of point to point orientations') isn't used elsewhere, and so is a bit opaque. The wording in the text, ("We...quantify cluster directionality by measuring the interquartile range of the orientations of all specimens relative to each other...") makes is sound as though clusters are defined by the orientation of individual fossils, whereas elsewhere it more sounds as though isotropy is being used to describe

the outline of the community on the bedding plane (something that would make more sense in context of the fluid dynamics argument invoked on line 76). In sum, I think the methods behind the identification and measurement of clusters needs much better explanation.

We apologize for this lack of clarity. We measure the directionality by summing up all of the individual point to point orientations, namely the orientations of each specimen to each of all the other specimens, within a given radius. We have clarified this definition in the text on Lines 111-119:

> "We developed a metric to quantify cluster directionality by first measuring orientation between each point (here fossil specimen) to all the other points (fossil specimens), then defining the relative isotropy as the variability of these orientations, measured as the interquartile range (i.e. isotropy is measured by the interquartile range of all of the point-to-point orientations across the population). This directionality measure was visualised by plotting each of these point-to-point orientation measurements as a rose diagram, with isotropy indicated by small values, i.e. round isotropy plots, (Fig. 2 b,d,g), and anisotropy by high values, i.e. ovate isotropy plots (Fig. 2e,f,h,i)."

And Lines 675-690:

> "Isotropy (directionality of clusters) of the spatial distributions was quantified using the spatstat package[72] in R, by calculating the interquartile range of the orientations between every point within a population and every other point within a given radius[73]. These data are plotted as rose diagrams (Fig. 3 and examples in Fig. 2), where isotropy is indicated by only small variations (i.e. interquartile range) in the orientations, namely a relatively circular diagram. Strongly anisotropic distributions are highly non-circular, i.e. exhibit large variations."

Figure 2. – I also found this figure slightly confusing; the logic in the caption is well explained (if members of two different species are spaced closer together than individuals belonging to either of the species themselves, then this suggests inter-specific competition at smaller scales than intra-specific competition), but there are some slight mismatches with the text. For example, the text states, "the largest heteromyopic difference…was with Fractofusus and Charniodiscus procerus…where the inter-specific segregation occurred 1.21m before the intra-specific segregation of Charniodiscus procerus, and 2.85m than Fractofusus" (line 152)…however…looking at the plot, I can't make these measurements match up with what's shown on that line. It would perhaps help if the authors could highlight species pairs in the y-axis where heteromyopia is detected, and perhaps arrow specific examples that are highlighted in the text.

We appreciate that our figure was very unclear, so we have implemented two changes. Firstly, we have created a new Figure 4 which has the PCFs as well as barplots beneath them to illustrate non-CSR spatial patterns. The figure contains the five heteromyopic bivariate PCFs plots with the univariate species PCFs. Lines 212-217. Because visualising overlapping simulation envelopes makes it hard to read the plots, we have also included Extended data Figures 1 and 2 to include a individual plots for each population with simulation envelopes for all the bivariate PCFs (and their associated univariate PCFs) which show segregation.

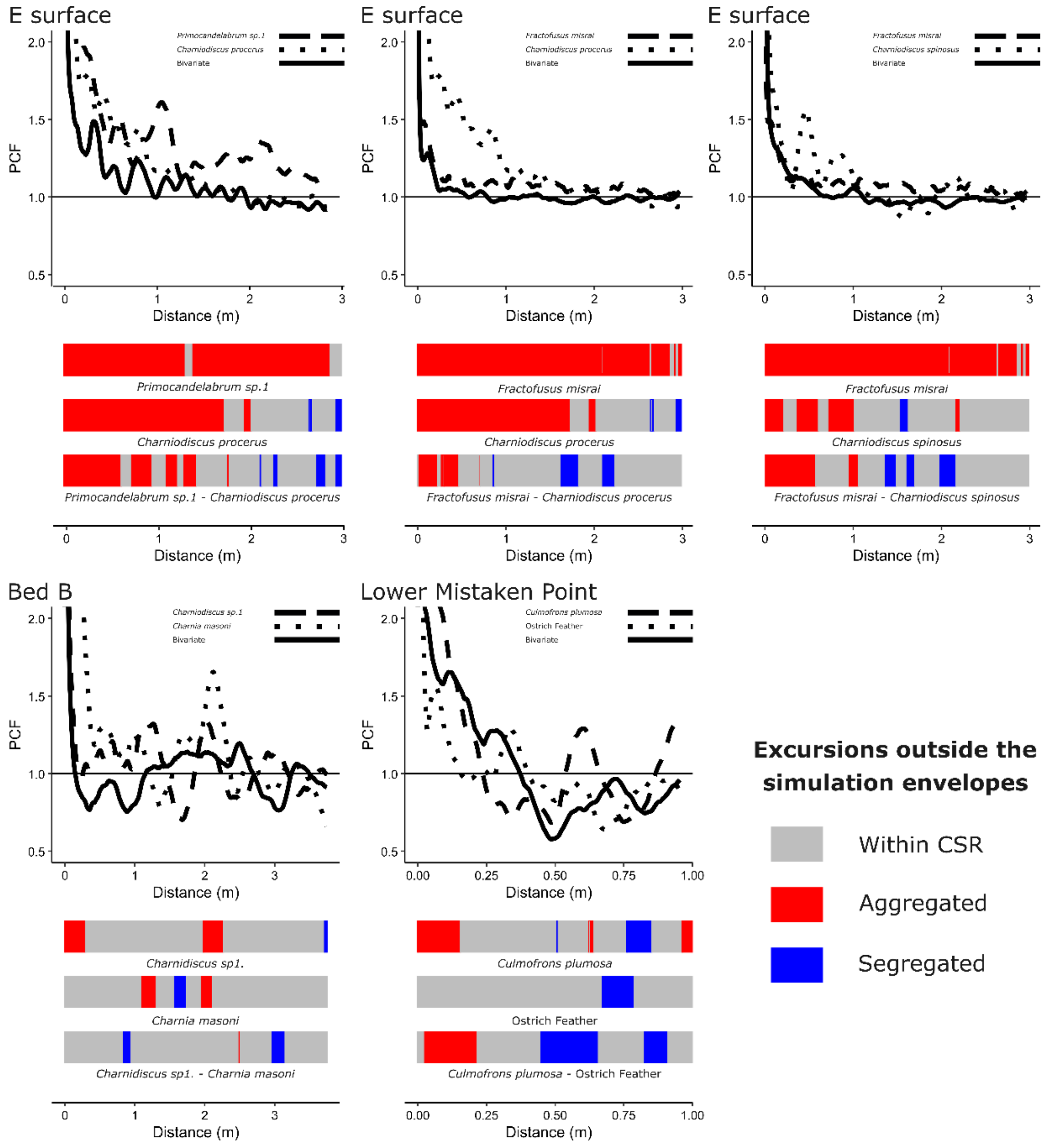

"Figure 4: PCF for mapped taxa pairs that exhibit inter-specific segregation. For all plots the x-axis is the inter-point distance between organisms in meters. The y-axis PCF=1 indicate CSR, <1 indicates segregation, and >1 indicates aggregation with the bars below the plots indicating whether the PCF is significantly > 1 (red), < 1 (blue) or CSR (grey). Heteromyopia is where the segregation (blue in the bottom bar) occurs at a smaller spatial scale (smaller x-axis value) in the inter-specific bar than in the univariate bars."

Lines 888-901:

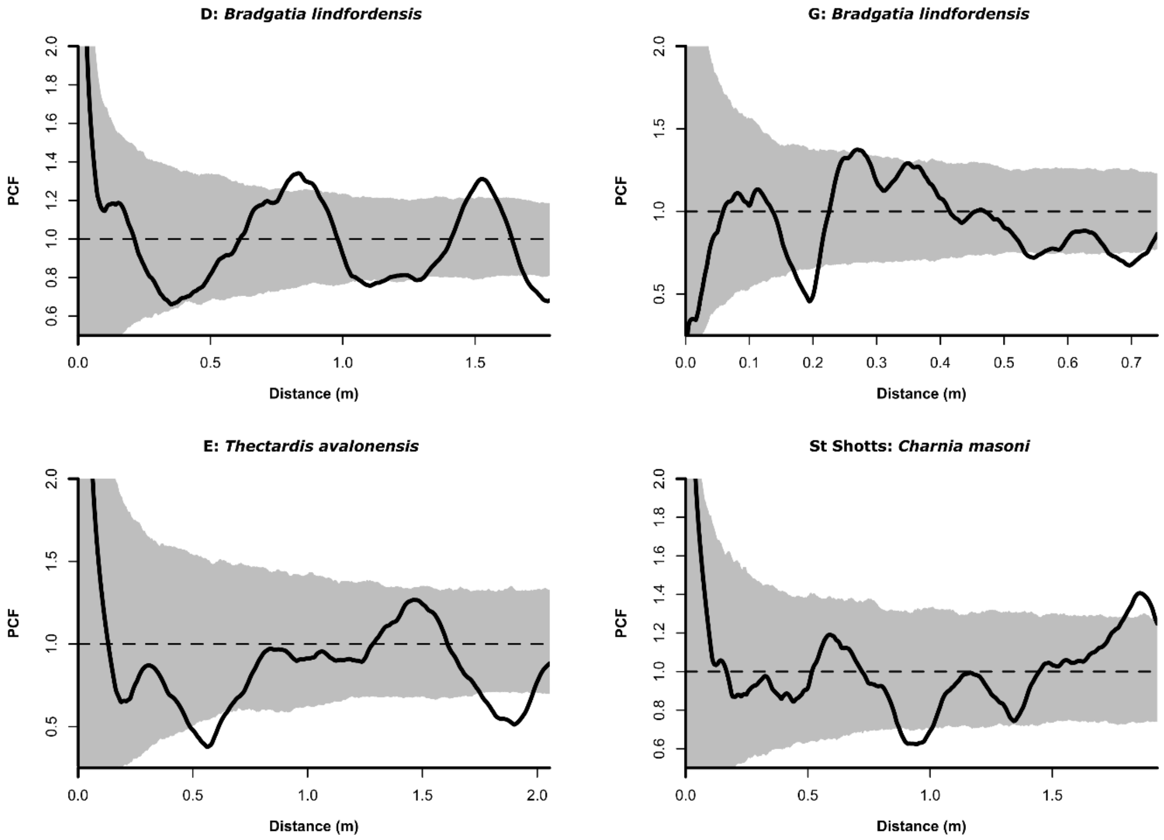

Extended data figure 1: PCF for mapped taxa that exhibit inter-specific segregation but no bivariate segregation. For all plots the x-axis is the inter-point distance between organisms in meters. The y-axis PCF=1 indicate CSR, <1 indicates segregation, and >1 indicates aggregation. Grey shaded area depicts the bounds of 999 Monte Carlo simulations of CSR, with non-CSR patterns indicated by PCF curves are not completely within these areas. The solid black line is the observed PCF.

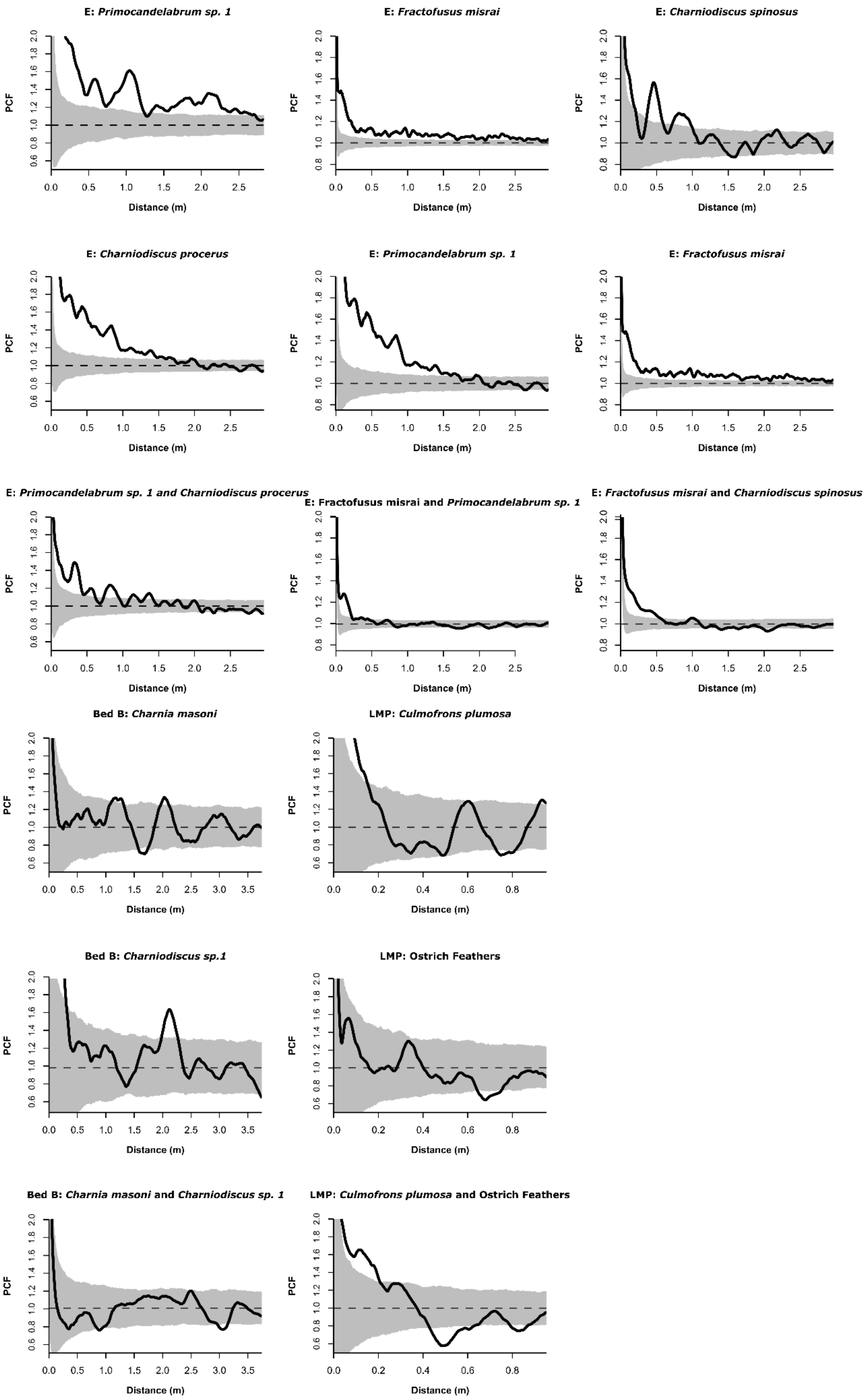
E: Primocandelabrum sp. 1
E: Fractofusus misrai
E: Charniodiscus spinosus
E: Charniodiscus procerus
E: Primocandelabrum sp. 1
E: Fractofusus misrai
E: Primocandelabrum sp. 1 and Charniodiscus procerus
E: Fractofusus misrai and Primocandelabrum sp. 1
E: Fractofusus misrai and Charniodiscus spinosus
Bed B: Charnia masoni
LMP: Culmofrons plumosa
Bed B: Charniodiscus sp.1
LMP: Ostrich Feathers
Bed B: Charnia masoni and Charniodiscus sp. 1
LMP: Culmofrons plumosa and Ostrich Feathers
PCF
Distance (m)

Extended data figure 2: PCF for mapped taxa pairs that exhibit inter-specific segregation. For all plots the x-axis is the inter-point distance between organisms in meters. The y-axis PCF=1 indicate CSR, <1 indicates segregation, and >1 indicates aggregation. Grey shaded area depicts the bounds of 999 Monte Carlo simulations of CSR, with non-CSR patterns indicated by PCF curves are not completely within these areas. The solid black line is the observed PCF.

Links to the 'second wave' of Ediacaran reproduction. – I'm a bit skeptical of the broader model being outlined here. On a specific level, the notion that evolving, and motile bilaterians may have broken stoloniferous connections in the subsurface is highly speculative, and is at odds with what we know about White Sea-aged environments and tracemakers. For a start, the microbial mat surfaces that characterize the White Sea (termed 'textured organic surfaces' by Tarhan and colleagues) are suggestive of thicker, more robust, and more complex mat communities than anything seen in the Avalon, and clearly presented a substantial barrier to both vertical and horizonal movement…what few ichnotaxa we have from this interval are either extremely small, or are limited to the top few mm of the mat surface (or conceivably, between shallow sediment laminae and the mat itself). Moreover, to the best of my knowledge nothing resembling a stolon has been identified from any surfaces beyond the Avalon, despite several (at least potentially) stoloniferous groups persisting into this interval. At a broader level, there's perhaps a hint of a circular argument being developed here…from the way the text is written, it makes it sound as though the breakage of stoloniferous connections by newly-evolving and motile metazoans, may have led to increased competition and thus the evolution of motile metazoans. Modes of reproduction cannot then be the only implicit driver - more complex and motile metazoans must thus have evolved first, for increasing competition as a driver of of evolutionary novelty to make sense. I don't mean to say that this model is wrong – it's an innovative idea and deserves to be out there – but, as currently framed, it doesn't come across as internally consistent, and doesn't match with any observations we have from the Avalon-White Sea transition.

We very much appreciate the reviewer pointing out this circularity and issues with this argument. Having re-thought out and investigated possible triggers for changes between asexual to sexual dominated reproduction, we think it is likely this change was triggered by a change in the levels of environmental homogeneity and stability in the Avalon compared to the White Sea, because facilitatively sexual organisms tend to be reproduce asexually in stable environments and asexual in more disturbed environments. Lines 324-354:

> "The lack of selection pressure for the majority of Avalonian taxa (due to this dispersal limitation) may explain the relative low rates of Avalonian diversification, with the majority of taxa not under strong evolutionary pressure, but the dominant taxa exerting a dispersal limited dynamic, thus enabling co-existence, but without a strong driver for natural selection. This co-existence dynamic would destabilise if there was a change in dominant reproductive strategy, from stoloniferous to water-borne, i.e. sexual reproduction (cf. Ref. 53). Facilitative sexual reproduction is common in extant sponges[54] and cnidarians[55] and also inferred in Avalonian taxa, for example with *Fractofusus*[16,56] and *Charnia*[24,57]. In extant animals, the relative dominance of asexual to sexual reproduction can change depending on the environment, with less stable, more disturbed habitats triggering higher levels of sexual reproduction[58]. The deep-sea Avalonian environment was relatively stable compared to the White Sea shallow water environment, with a more homogeneous background environment[59] and the death-events that killed and preserved the communities occurring around every 10-100 years, possibly up to every 1000 years[60–62], rather than much more frequently, ~ 2-3 times per year, in the White Sea environment[63,64]. This less-disturbed Avalonian environment is evidenced in their communities through mature communities present[21] and the older inferred maximum ages of specimens[65]. Therefore, the colonisation of the more disturbed shallow water environment, may have triggered a switch from asexual dominated communities, to sexual dominated communities. The increase in sexual, i.e. waterborne reproduction, and its associated reduction in dispersal limitation, increases competition and selection pressure for both the

previously dispersal limited animals as well as the weaker competitors that had previously been shielded by the dispersal limited dominant taxa. Thus, increase in Ediacaran diversity around 550 Ma[9] has the potential to be explained from the increase of dispersal distances[66] and subsequent changes of selection pressure that comes with a transition from wide-spread stoloniferous reproduction, to systems dominated by waterborne propagation[53,6667] and thus the prior co-existence mechanism."

While we appreciate that preserved stolon are very rare in the White Sea assemblage, but because the earliest diverging animals are sponges, cnidarians and ctenophores all of which include coloniality and clonal reproduction (see e.g. Hiebert et al. 2021, Fig. 1), it is parsimonious to assume that colonial reproduction was present in the White Sea assemblage, given the widespread coloniality of basal animals (West et al. 2015, Hiebert et al. 2021)

S.A. West, R.M. Fisher, A. Gardner, & E.T. Kiers, Major evolutionary transitions in individuality, Proc. Natl. Acad. Sci. U.S.A. 112 (33) 10112-10119, (2015).

Hiebert LS, Simpson C, Tiozzo S. Coloniality, clonality, and modularity in animals: The elephant in the room. J Exp Zool (Mol Dev Evol). 2021; 336: 198–211.

Reviewer #2):

I really loved reading this work by Mitchell and Manica. This manuscript is quite an interesting and well-structured paper that proposes an ecological mechanism— heteromyopia driven by stoloniferous reproduction— to explain key macroevolutionary patterns in early animal diversification during the Ediacaran period.

The work presents a novel and plausible hypothesis, supported by quantitative spatial analyses of Ediacaran fossil assemblages and mechanistic modeling. It tackles a significant question: what controlled the tempo and mode of early animal diversification? The authors argue convincingly that the prevalent mode of reproduction (stoloniferous asexual reproduction) in early Ediacaran (Avalonian) communities led to a peculiar competitive dynamic called heteromyopia, which in turn limited selection pressures and constrained diversification until a shift towards sexual reproduction and/or increased mobility occurred, heralding the "Second Wave" of Ediacaran diversification.

Although there are assumptions about certain aspects of reproductive mode and other ecological factors at play, I think the arguments put forward in this manuscript are convincing and am happy to recommend this paper be accepted with some very minor changes and suggested edits, which are more like copyediting issues (noted below). I would like to congratulate the authors on writing such a compelling manuscript.

Thank you so much for your enthusiasm for our work. We hope that the revisions made in response to the other reviewers will help to clarify our assumptions and we appreciate the attention to detail given by the points you have noted below.

Potential Copyediting Errors & Minor Issues:

• Page 2, Line 41: "in the Avalon there are instances" -> maybe to make clearer "in Avalonian communities there are instances" or "within Avalon assemblages there are instances" for consistency with other phrasing.

Changed on Lines 41-42:

"However, in Avalonian communities there are instances.."

• *Page 4, Line 74: "that is clusters which do not have any directionality, i.e. are circular" -> "that is, clusters that do not have any directionality (i.e., are circular..." - Minor punctuation.*

Brackets added on lines 88-89:

> "This attachment results in isotropic clusters, that is clusters which do not have any directionality, (i.e. are circular) ."

• *Page 5, Line 114: very minor: "segregation up until (or over) the point where the observed data crosses PCF=138." - "up to" is sometimes preferred over "up until."*

Changed up until -> up to on Line 151

• *Page 6, Line 121: "In contrast, the Bristy Cove Fractofusus showed high levels of directionality, likely due to the relative immaturity of the community (as inferred by the small body sizes and large percentage in the smallest size class, Table 1), suggesting that the population had not enough time to reproduce." -> "...suggesting that the population had not had enough time to reproduce" or "...suggesting the population lacked sufficient time to reproduce."*

Changed on Lines 163-4:

> "...suggesting that the population lacked sufficient time to reproduce..."

• *Page 8, Line 163: "Heteromyopic relationships were present if the interspecific estimate (purple point) was found at smaller minimum distance than either of the intra-specific once (blue/red)." -> "...than either of the intra-specific ones (blue/red)."*

We have changed the associate figure and caption, so this line is no longer present.

• *Page 10, Line 191: "smaller spatial scales than intra-specific competition for one taxon indicating" -> "smaller spatial scales than intra-specific competition for one taxon, indicating..." (comma for clarity).*

Comma added on Line 271.

• *Page 12, Line 252: "increase in Ediacaran diversity around 550 Ma7. has the potential be explained" -> "...has the potential to be explained..."*

Added "to" on line 350

• *Page 13, Line 277: "7.924 Thus, our mechanistic model..." - The "7,924" seems like a stray citation number or error. Did you mean 7, 9 and 24?*

Thank you for noticing this, we have now updated and corrected this reference on Line 387.

• *Source 213, Page 23, Line 481 (Figure 2 Caption): "Minimum distance of segregation" - "minimum" should not be capitalized unless it's part of a formal name.*

Thank you for this note. We have changed the figures such that this mistake is no longer present.

• *Page 24, Line 504: "where isotropy was indicated by only small variations" -> "where isotropy is indicated by only small variations" (present tense for describing a general method/interpretation).*

We have changed the tense (was to is) on line 680.

Our apologies for our mistakes with the tense. We have gone through the manuscript and methods and updated to ensure the correct tense for the methods and interpretations.

• *Page 25, Line 532: "For all populations--(,)-- the number of normally distributed cohorts within the populations were found 64" -> "...within the populations was found"*

Thank you, we implemented this change on Lines 719-720.

• *Page 26, Line 558: "Thomas clusters are associated with reproductive processes 14,32,38,66 so a Thomas Cluster sizes are good way" -> "...so Thomas Cluster sizes are a good way..."*

We have modified this text in response to Reviewer 3, so this change is no longer needed.

• *Page 27, Line 595: "aavalon=20; awhitesesa=36; anama=28" -> "whitesesa" is meant to be "White Sea"*

Thank you, we have changed whitesesa to White Sea on line 846.

• *Page 29, Line 605: "Note that some cases, such as the G surface Bradgatia where Thomas Clusters are not a good model fit, the cluster size tends to zero." -> "...Bradgatia, where Thomas Clusters are not a good model fit, the cluster size tends towards zero." (please note the use of commas elsewhere as well)*

Due to our updated analyses we have modified this text, with suitable punctuation as suggested.

Note that where populations didn't exhibit aggregations, such as the G surface Bradgatia and Bristy Cove Fractofusus, fitting cluster models was not appropriate, so were excluded from these analyses.

Reviewer #3 :

This manuscript uses data on the spatial distribution of in-situ, sessile benthic organisms that have been preserved as fossils to derive hypotheses to explain the burst of rapid diversification of the Ediacaran animal communities after a longer period of little change. The idea is that widespread reproduction via stolon in the first animals led to dispersal limitation of the dominant species and coexistence of weaker competitors operating under reduced selection pressure. The hypothesis is that disappearance of the stoloniferous reproductive strategy should increasing competition and selection pressure and lead to the observed burst of diversification (the Second Wave). The hypotheses derived from the reanalysis of mapped fossil communities are then tested by simulations of a metacommunity model.

I find the possibility to obtain and analyse maps of organisms some 500 million years ago fascinating, and the idea to explain the burst of diversification of the Second Wave by changes in the spatial structure of the communities (driven by changes in the reproductive strategy) very interesting. However, my impression is that the evidence presented in favour of the hypotheses is not very strong. Parts of the spatial analysis (the determination of the scales of segregation) are a bit weak and the mechanistic model is not very convincing since it seems not to match the scales and the biology of the system (and the data extracted from the fossil maps) very well. A specifically tailored spatial model that can generate spatial data that can be directly compared to that extracted from the fossils (and includes stolon reproduction vs. water-borne reproduction) would be needed to provide more conclusive evidence for the interesting hypothesis.

We thank the reviewer for their interest in this paper. We appreciate the concerns about the methodology, and as detailed below, have provided evidence strengthening our conclusions. We have also (as detailed below) expanded out on our discussion and how to interpret our mechanistic model to explain the links between the spatial scales of the data and model better. We think that part of this lack of clarity was how we presented our mechanistic model in terms of heteromyopia, when more precisely, it was modelling dispersal limitation, which is the process underlying heteromyopia patterns. With these clarifications we feel that the models and statistical evidence sufficiently support our conclusions, without bringing in a new model approach.

lines 18-21, 24-28 This is an interesting hypothesis

lines 82-95 This is an interesting analysis.

Thank for your interest in our hypothesis and analyses.

lines 75-79 Perhaps you could show two contrasting spatial maps (circular vs. elongated clusters) of the organisms to illustrate the dispersal clusters?

We agree better visualisations are needed, so have added a new Figure 2 showing simulated and extant observed isotropic and anisotropic patterns (Fig. 2), and gave more detail about how they should be understood on Lines 111-119:

> "We developed a metric to quantify cluster directionality by first measuring orientation between each point (here fossil specimen) to all the other points (fossil specimens), then defining the relative isotropy as the variability of these orientations, measured as the interquartile range (i.e. isotropy is measured by the interquartile range of all of the point-to-point orientations across the population). This directionality measure was visualised by plotting each of these point-to-point orientation measurements as a rose diagram, with isotropy indicated by small values, i.e. round isotropy plots, (Fig. 2 b,d,g), and anisotropy by high values, i.e. ovate isotropy plots (Fig. 2e,f,h,i).

And on lines: 687-697:

> "We first simulated Thomas Cluster distributions (Fig. 2a), with no inherent directionality, which showed a relatively isotropic, namely circular rose diagram (Fig. 2d). We then applied a 2x transformation to these Thomas Cluster points along the x-axis only (Fig. 2b), which showed an ovate rose diagram (Fig. 2e), with a 3x transformation (Fig. 2c), showing a more elongated rose plot (Fig. 2f). To illustrate how IQR varies with different reproductive modes, we ran our analyses on three observed examples. First, an extant hydroid stoloniferous colony[74] to demonstrate the high isotropy of stoloniferous reproduction (Fig. 2g), and then for the sponge *Advhena magnifica* in two areas of an extant deep-sea coral and sponge community[30] exhibiting high anisotropy, with the anisotropy varying due to different current direction on the two different areas (Fig. 2h,i)."

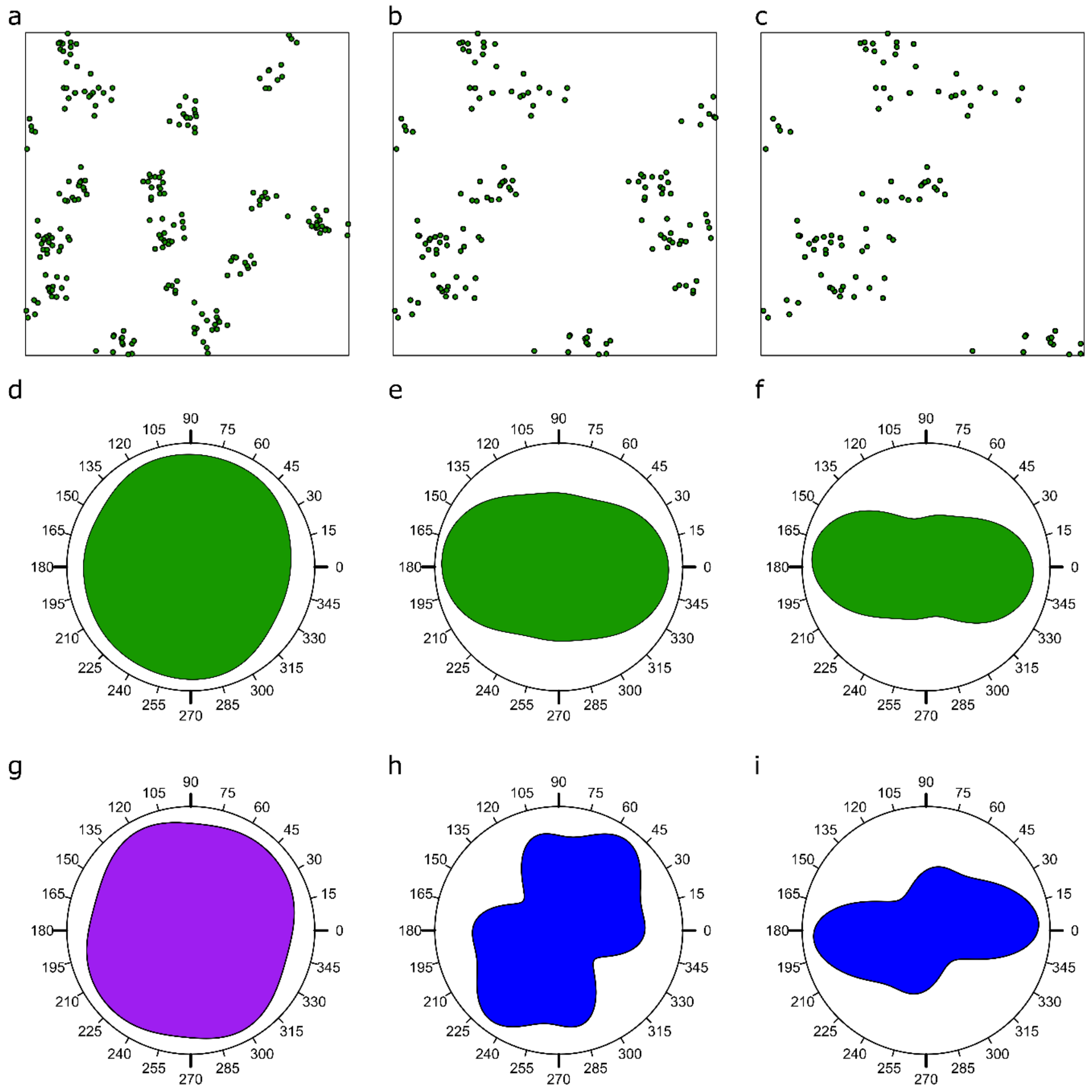

"Figure 2: Examples of different levels of isotropy. **a)** Simulated Thomas clusters with their associated isotropy plot (as one would expect for clonal organisms) **d). b)** Shows the same Thomas Clusters with a 2x directionality on the x-axis (as might be caused by broadcast spawning), shown by the elongated isotropy plot in **e)**. **c)** shows the simulated Thomas Clusters with a 3x directionality, and an associated more ovate isotropy plot in **f)**. **g)** observed examples of a hydroid colony connected by stolon, and for the deep-sea sponge *Advhena magnifica* in two sections of a deep sea coral and sponge community subject to different current directions in **h)** and **i)** (from Ref. 30).

line 88 What is the first and second factor?

Our apologies for this mistake. We have updated the text on Lines 119-124 to make it clear what the three factors are.

"There are three factors that can impact the size of dispersal clusters. First, stoloniferous connection between individuals will limit the size of dispersal clusters, since water-borne propagules travel further than connected ones[31–34], leading to smaller cluster sizes. Secondly. because such reproductive cluster sizes depend on the height of the parent, here we consider

normalised clusters (i.e. we divide the cluster size by the maximum specimen height of population) rather than absolute sizes. The third factor ...”

line s 106-114, 140-156 Looking at the graphs of Extended data Fig. 2 it looks that you have some issues with too small bandwidths, which lead to somewhat rugged pair correlation functions. This makes your objective to determine scales of segregation difficult to attain since some of the fluctuations of the function may be due to stochastic noise. Given that competition is a more cumulative mechanisms, you may use instead a variant of the K-function(K(r)/pi r^2), which is the direct cumulative analogue to the pair correlation function: it gives the mean (normalized) neighbourhood density of points within distance r of the focal individuals). This version of the K-function has no issue with the bandwidth and will produce smoother estimates. Given that the width of the simulation envelopes depends very much on the number of individuals of the patterns (it is basically proportionally to 1/N), you may also consider to just look at the distances where the empirical summary functions has values below 1 and use the lowest value within a competitive neighbourhood as measure of strength of competition. line 170-179 I am curious to see what you get out using a more stable summary function to estimate segregation.

We very much appreciate how the PCFs can look very noisy. We do not think the K function is appropriate for this work because it is a cumulative function so that a high aggregation at small spatial scales would cancel out segregation at larger spatial scales. Therefore, this cumulation would mask both the spatial scales and magnitudes of any segregation. The PCF is related to the K function as follows, so focusses better on how patterns change at different spatial scales:

$$PCF(r) = \frac{K'(r)}{2\pi r}$$

It can be hard to determine an appropriate bandwidth/smoothing factor for a given PCF since it would depend on sample size, density and the underlying pattern, and most bandwidth calculations only consider the density – for example we could imagine having 30 points within 1 m^2 would give a very noisy PCF, with a wide simulation envelope, but the same underlying pattern but over 100 $m^2$and 3000 points would have the same bandwidth, but be much smoother. While the bandwidth can be manually adjusted to get an appropriate level of smoothing (assessed visually), we thought it more reproducible to provide a single approach for all populations, while acknowledging that this treatment will result in different levels of noise.

We have used as default the general kernel which is $bw = \frac{c}{\sqrt{5\lambda}}$ where λ is the point density and *c* is between 0.1 and 0.2, as recommended by Stoyan and Stoyan 1994, and used in spatstat (Baddeley et al. 2015).

To explore the impact of different bandwidths (which is inversely proportion to the smoothing applied) on our result, we varied the bandwidth from 0.01 (corresponding to the smallest bandwidth which is c = 0.1 and the lowest population density of *Beothukis* on E surface, which would be $bw_{E\ beothukis}$ = 0.007) and the highest bandwidth of 0.15 (corresponding to the largest bandwidth which is to a c = 0.2 and the highest population density of *Fractofusus* on H14 surface, which would be $bw_{H14\ fractofusus}$= 0.0139) (bw = default, 0.01; 0.05; 0.10; 0.15).

Besides sensitivity analyses of the smoothing factor, we have further interrogated the robustness of our regression throughout by repeating a series of regressions with different permutations.

1) Using only populations with segregations under 1 are considered.
2) With a range of different distances within which we look for segregation
    a. All (as in the main text)
    b. Above 1% of total distanceto account for edge effects, under 5% to account small-scale segregations possibly due to finite body size (~10-20cm).

c. Between 5% and 95%, which focusses on the range which would exclude the instabilities at the edges of the spatial ranges calculated.

Across all permutations, the median *p*-value was 0.0013,, maximum 0.0097, and median Adjusted R^2=0.4938, minimum R^2=0.3464, maximum R^2 = 0.5652.

We have added these new analyses on Lines 780-798.

> *"Sensitivity analyses*
>
> In terms of the input variables for our regression, there are three different sets of permutations that can impact the PCF, so influence the minimum PCF value: 1) The range of PCF distances (r) because the calculated PCFs near r = 0 and near the edge of the distance range can be unstable. 2) The PCF is calculated using a smoothing estimator to account for noisy data. We have used as default the kernel bandwidth $\mathrm{bw} = \frac{c}{\sqrt{5\lambda}}$ where λ is the point density and c is between 0.1 and 0.2, with the default in spatstat set to c = 0.15[73,82]. The larger the bandwidth, the more smoothing is applied, so the signal is less noisy. 3) Whether we consider all spatial patterns or only the ones which have PCF < 1, i.e. exhibit some spatial segregation. In order to test the impact of changing these parameters on our regressions we calculated 24 different permutations for the PCF to find the minimum PCF as follows: 4 sets of different distance ranges: 1) All distances, excluding the very small (< 1% of maximum distance), excluding the small distances that likely overlap with the body-sizes of the specimens (~20cm < 5%), and excluding the small and very large (5% < r < 95%). For each distance permutation we calculated the PCF for three different levels of bandwidth, less smooth (c = 0.1); default/medium (c=0.15) and smoother (c=0.2). Then for each permutation of distance and smoothing we input 1) all the populations and 2) only the populations than exhibited PCF < 1 over the distance ranges and smoothing considered."

And added them to the Extended data section as Table 4:

Range of PCF distances	Band width for smoothing	Minimum PCF value	R^2	Adj. R^2	*p*-value	AIC
all	0.1	all	0.4705	0.4393	0.0012	-11.36
> 1%	0.1	all	0.5154	0.4869	0.0005	-12.62
> 5%	0.1	all	0.5774	0.5525	0.0002	-13.74
5% < r < 95%	0.1	all	0.5774	0.5525	0.0002	-13.74
all	0.15	all	0.5894	0.5652	0.0001	-23.96
> 1%	0.15	all	0.5815	0.5569	0.0001	-23.30
> 5%	0.15	all	0.5815	0.5569	0.0001	-23.30
5% < r < 95%	0.15	all	0.5765	0.5516	0.0002	-22.64
all	0.2	all	0.5638	0.5381	0.0002	-28.22
> 1%	0.2	all	0.5575	0.5315	0.0002	-28.01
> 5%	0.2	all	0.5575	0.5315	0.0002	-28.01
5% < r < 95%	0.2	all	0.5430	0.5162	0.0003	-26.31
all	0.1	<1	0.3900	0.3464	0.0097	-9.85
> 1%	0.1	<1	0.4417	0.4019	0.0050	-11.43
> 5%	0.1	<1	0.5120	0.4771	0.0018	-10.87
5% < r < 95%	0.1	<1	0.5120	0.4771	0.0018	-10.87
all	0.15	<1	0.5288	0.4952	0.0014	-19.69
> 1%	0.15	<1	0.5276	0.4938	0.0014	-19.80
> 5%	0.15	<1	0.5276	0.4938	0.0014	-19.80
5% < r < 95%	0.15	<1	0.5276	0.4938	0.0014	-19.80

all	0.2	<1	0.4987	0.4629	0.0022	-23.25
> 1%	0.2	<1	0.4972	0.4613	0.0023	-23.60
> 5%	0.2	<1	0.4972	0.4613	0.0023	-23.60
5% < r < 95%	0.2	<1	0.4970	0.4610	0.0023	-23.59

In terms of smoothing, all bandwidths correspond to the significant and strong linear relationships (high R^2 values). As such, we think that leaving the default settings for this bandwidth, with the appropriate default use of c, is the most unbiased way to present our data.

These results demonstrate the robustness of our results. In terms of the most appropriate permutation of variables we would prefer to present the results which do not have any filtering of the input data, since such filtering could have the potential to bring in biases. However, if the reviewer feels differently, we could go with the permutation that they find most appropriate.

Figure 3 The minimal value of the pair correlation function segregation is zero, which indicates maximum segregation (i.e., no neighbour at the given distance). Therefore, the y-axis with values < 0 does not make sense. The range of values in the regression analysis must be constrained to >=0.

Our apologies for this confusion. We prepared this plot using the metric of 1-PCF_{min} to add comparisons with the regression analyses, but appreciate that it was very confusing. We have updated the figure to be more explicit on lines 244-248, and to aid further clarity we have broken it down in terms of predictor variables.

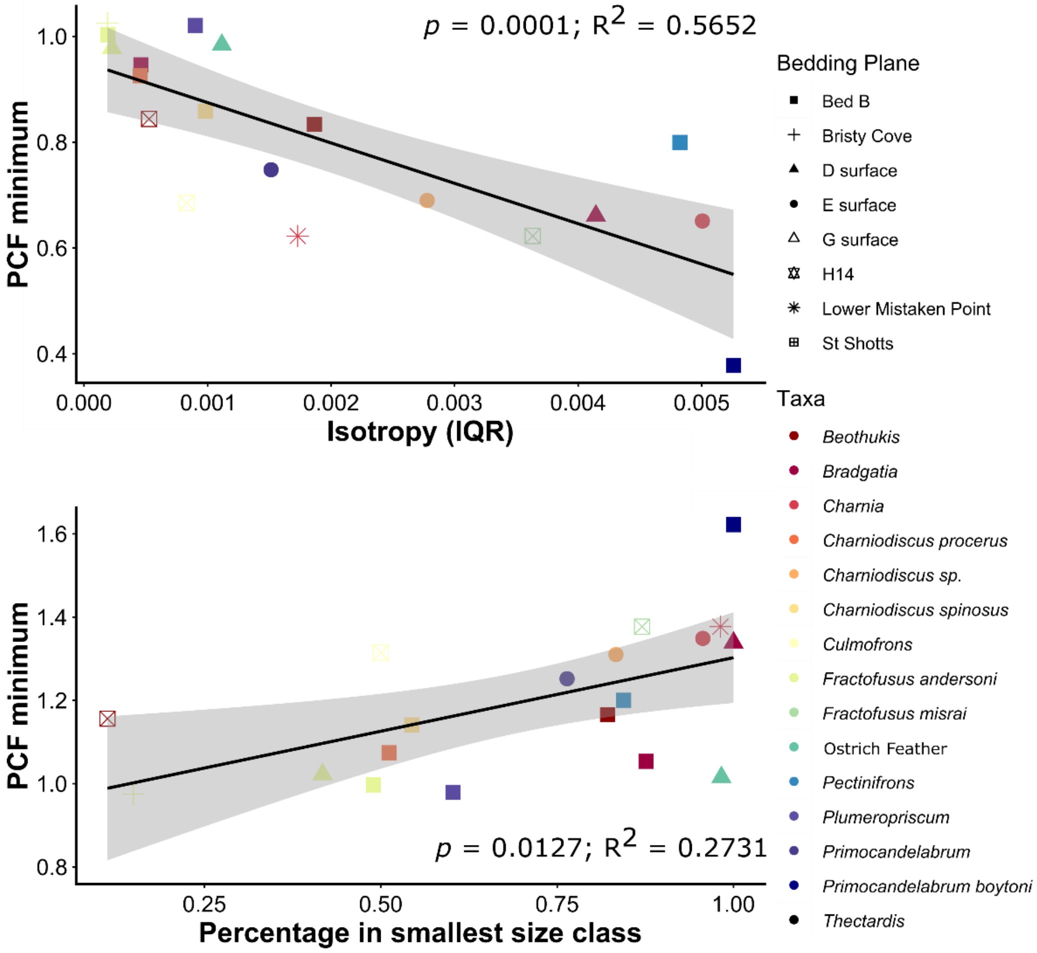

"Figure 5: Relationship between predictive variables with the most power and the minimum PCF. Black line shows the best fit for each regression is given and the grey area is the 95% confidence interval.

line 264 It is unclear how the mechanistic model incorporates heteromyopia at the scales extracted from the spatial analysis."

line s 273-277 If the model captures the mechanisms occurring at the three assemblages…

Our apologies, we didn't present this model very clearly. The mechanistic model includes dispersal limitation, so doesn't model heteromyopia directly,. Instead, heteromyopia is a consequence of high dispersal limitation. In terms of how the model connects to our analyses, we have explained this on lines 366-375:

"In this mechanistic model, dispersal was modelled as the relative percentage of specimens after a reproductive event that left the local community, i.e. joined the rest of the metacommunity, such as where dispersal parameter ω < 1.0, 100% of new specimens would remain inside the community, and ω > 1.0 reflects where specimens emigrate. As such, ω represents the degree of dispersal limitation. For example, if all specimens were reproducing via stolon, we would expect ω << 1.0 since none would be waterborne, so they wouldn't be able to leave their community. In contrast if all specimens were reproducing via spawning, they would be carried in the water, so a large proportion would leave their natal community."

We have clarified that the model includes only dispersal limitation explicitly (so heteromyopia implicitly) throughout the manuscript by changing heteromyopia -> dispersal limitation where appropriate on Lines 248, 259, 273, 274, 279,283, 303, 317.

Figure 4 Although the Avalon posterior distribution has a peak at low dispersal parameters, all three distributions are quite flat. The dispersal parameter need to be explained in the main text.

We agree that the dispersal parameter is relatively flat. However, since there also are clear peaks between the three assemblages, there is still a clear signal.

We have expanded on what the posterior distributions lines 380-382.

"Whilst the posteriors were broad (as one would expect given the limited number of available communities) the shape differs among the three assemblages, showing clear increase from the Avalon to the White Sea, and a decrease in the Nama."

line 445-446 There is something wrong with the reference.

Thank you for catching this, we have now corrected and updated this reference.

line 504 Fig. 1?

Thank you for catching this, we have now corrected and updated this reference to Fig. 3.

Line s 531-535 Unclear what you did and why you did it.

Our apologies for the lack of clarity. We have explained our approach in more detail on Lines 711-716:

"The previous SPPA was published across four different studies[16–19] so to ensure that the data was analysed consistently, all intra and inter-specific spatial distributions were (re)analysed as described below. These SPPA provided the data needed for the regression analyses, namely the presence or absence of aggregations and segregations, the segregation strength as measured by the value of minimum PCF values and the size of cluster radius"

Line s 547-552 How did you correct for the non-rectangular shape of the observation area? What is the pd value? See also my comments about the rugged pair correlation functions above. line n556 You should briefly explain the Thomas process and how it can be used to determine the cluster size.

Our apologies for the lack of clarity in our methods. We have added a section on edge correction on Lines 705-704:

> "The mapped areas are irregularly shaped, so to account for edge effects, Ripley's isotropic edge correction weight is used[78], whereby the number of points within an incomplete circle are scaled by the proportion of the area of the circle that lies within the mapped area[77]. "

We have clarified our methodology including Thomas clusters/processes on Lines 734-736.

> "To quantify the spatial distributions and find any significant deviations from CSR, pair correlation functions (PCF) are used[44]. The spatial scales and magnitudes of non-random spatial patterns are determined by running 999 Monte Carlo simulations for each species, on a homogeneous background, to generate simulation envelopes around the random (PCF = 1). Homogeneous backgrounds were used because previous work has established the lack of significant influences by heterogeneous backgrounds for these Avalonian communities[19], so such heterogeneities do not need to be taken into account, in contrast to many modern systems[40,79]. The highest and lowest 5% of simulations are excluded from the simulation envelope to exclude outliers[77]. Where segregation (PCF < 1) fell outside the Monte Carlo envelope, the minimum PCF value is recorded. So that larger values indicate more segregation[44], the metric ($1-PCF_{Min}$) is used. Reproductive processes can be modelled spatially using Thomas Cluster models[16,38,44,7744,77], which are parametrized by the number of clusters in a given area (i.e. the density of the clusters), the density of points (here specimens) within a cluster, and the radius of the clusters. The density of the points within the cluster follows a Gaussian distribution such that the highest density is in the center of the cluster, i.e. around the presumed parent. The number of "offspring" in a cluster is therefore a function of the density within the cluster and the size of the cluster. Thomas Cluster models are fitted to the small scale PCF to determine the best-fit cluster size for the distribution[72,80–82]. Thomas clusters [16,38,44,77] have been established to be the dominant mode of aggregation for Avalonian communities[19], so are appropriate for this modelling.

line s 576-599 The model should be explained in more detail. Looking at ref. 54 it is unclear how this model fits your assumptions/hypotheses. You base your hypotheses on neighbourhood analyses of individual organisms (and stolon reproduction), whereas the model is a metacommunity model that looks at patches (of local communities), connected by dispersal. Your data represent maps of individuals within a certain area, which may correspond to single patches of the metacommunity model. Additionally, your hypothesized distance of dispersal are rather inside the patch than between patches as assumed in the metacommunity model.

We agree that the model wasn't well explained in the text. We have updated the text to explain the dispersal parameter on lines 349-357. For this model, the dispersal parameter does account for dispersal only inside the patch, with a varying amount of possible dispersal outside the patch (if $\omega >$ 1.0). It is this balance between inside (i.e. dispersal limited) and outside (i.e. more widespread/waterborne) that we are testing.

We have updated the methods section to qualitatively explain the model in more detail to ensure that all readers, regardless of their field, have an understanding of the parameters. The mathematical description is left as a reference to the original paper. Lines 804-821.

> "Within this mechanistic model framework, a reproductive event followed by dispersal are simulated, then the alpha diversity across the total number of communities is calculated. Full

mathematical details are given in Ref. 68. Qualitatively, the reproduction rate depends on the niche overlap and the environmental condition (parameterized between zero and one for each community) within a community. The niche overlap h is the variance of the Gaussian distribution that describes how much a given species overlaps with another, such as high h corresponds to more niche overlap, and low h to a more specialised niche. Dispersal is assumed to be passive (such as through corals spawning), with the dispersal parameter as the relative percentage of specimens after a simulated reproductive and then dispersal event than remain within the community, versus leave the local community and so join the rest of the metacommunity. Where dispersal parameter $\omega < 1.0$, all new individuals remain inside the community, and $\omega > 1.0$ reflects where individuals leave the local community and join the rest of the metacommunity. For example, if all specimens were reproducing via stolon, we would expect $\omega << 1.0$ since none would be waterborne, so they wouldn't be able to leave their community. In contrast if all specimens were reproducing via spawning, they would be carried in the water, so the majority would leave their natal community and join the wider metacommunity."

We would like to thank their reviewers for their attention to details, and their reviews for really approving our manuscript.

We have implemented the suggestions of the editor, including shortening the abstract and making it more accessible to a non-technical reader:

Abstract

The appearance of the oldest known animals during the late Ediacaran (~574 Ma) was followed by a phase of slow diversification, until a later burst of rapid diversification known as the Ediacaran "Second Wave". The reasons behind the tempo of diversification are poorly understood. Here we investigate how reproductive mode mediated community dynamics, and in turn Ediacaran macroevolutionary change. We show that widespread reproduction via stolon (namely via filaments connecting clones) limited within-species competition, leading to between-species competition acting at smaller-spatial scales than within-species competition, a phenomenon called heteromyopia. Heteromyopia enables co-existence of sub-optimal competitors because the dispersal limitation of the dominant species limits the occupation of the optimal habitat, so that lesser competitors can still exist within the same community, operating under reduced selection pressure. We explored the consequences of this dispersal limitation on community diversity using a mechanistic model showing that the change from stoloniferous to sexual reproduction that coincided with the Second Wave could explain the sudden increase in diversity observed in the fossil record. We conclude that widespread asexual reproduction via stolon likely constrained early animal evolution, limiting diversification until the onset of widespread sexual reproduction.

All line numbers refer to the tracked changes version.

Reviewer #1 (Remarks to the Author):

The authors have done a nice job with this revision – I find the text much easier to follow, the new figures do a great job of illustrating the patterns in an intuitive way, and any lingering circularity has been removed. Barring a few, tiny typos and formatting errors, I think that this is now ready for publication, and I look forward to seeing it in print. These last few things are numbered below:

1. "Importantly, heteromyopic dynamics are hard to distinguish from neutral dynamics based on most ecological metrics, such as species area distributions [23]" (line 44); I believe the Darroch et al. paper analyzed species-abundance distributions, rather than species-area distributions.
2. Line 200; double comma.
3. Line 222; ditto.
4. Line 774; ditto.
5. "As such, our data is a good reflection of documented early animal life" (line 246); this same sentence is given in the first line of this section, and so I think here it's redundant.

Thank you catching these errors, which we have now addressed as suggested.

Reviewer #2 (Remarks to the Author):

According to my reading of the new version of the manuscript in the context of the comments by

other reviewers and mine, the authors have successfully addressed the conceptual and methodological critiques raised during the initial review phase.
I had initially overlooked the mobility problem raised by reviewer 1, and I appreciate the authors' trying to resolve it using an environmental stability argument. The original manuscript relied on a seemingly circular argument where motile metazoans were both the cause and the result of increased competition through stolon breakage. In response, the authors have now reframed the macroevolutionary trigger as a transition driven by environmental stability. This new framing is more biologically parsimonious and better reflects the geological record of these environments.
Methodologically, the authors also seem to provide a good response to Reviewer 3's concerns regarding the ruggedness of the Pair Correlation Functions (PCF) and potential stochastic noise. Rather than switching to the suggested K-function, which they argued would mask scale-specific segregation, the authors performed an exhaustive sensitivity analysis across different permutations of kernel bandwidths and distance ranges.
Furthermore, the addition of new figures, specifically Figure 1 for visualizing heteromyopia and Figure 2 for interpreting isotropic rose diagrams, directly resolves the opacity issues noted by all reviewers, providing a clear visual bridge between complex spatial point patterns and biological interpretations.
I am happy with this round of revisions and recommend the paper for acceptance.

Thank you, we really appreciate your comments.

Reviewer #3 (Remarks to the Author):

The authors responded well to my comments. The new figures are a great addition, and I am pleased with the sensitivity analysis, which increases confidence in the robustness of the approach. Overall, the manuscript now presents an intriguing hypothesis about changes in life many millions of years ago and provides solid evidence in its favour. This makes it really special.

Thank you so much for your comments, we really appreciate your help with our paper.

However, on the downside, by closely re-reading the manuscript I found several points where the authors could improve readability. For example, I would prefer the more traditional way of writing figure captions, where each panel is explained in turn (and each panel should be labelled and referred to in the text!). Also, technical terms are often inconsistent and confusing, with many different terms being used for one quantity. Additionally, some figures are of poor graphical quality and must be improved. Finally, paragraphs are often too long and should be split. All of these issues could be resolved with little effort.

Thank you for your comments and attention to detail. We have changed all the figure captions as you have suggested as given below, provided vector file for the figures to ensure good resolution and implemented your other suggestions as detailed below:

Figure 1 Schematic diagram demonstrating heteromyopia. The blue and black dots represent different species. **a)** If both species are stoloniferous, then all specimens within a cluster (given by

the circle), remained connected. In this instance, the spatial scales of competition would not be within the cluster (circle), but only between them, shown by the black arrowed lines in **b)**. However, competition can still occur between two different species, for example within the grey dashed box in **b)**, which would occur on spatial scales smaller than within species where the two clusters overlap (as seen in **a**), and shown by the red arrows in **c)**, which depicts the blue specimens that the two black specimens would compete with.

Figure 2: Examples of different levels of isotropy. **a)** Simulated Thomas clusters with their associated rose diagram (as one would expect for clonal organisms)**. b)** Shows the same Thomas Clusters with a 2x directionality on the x-axis (as might be caused by broadcast spawning) and **c)** shows the same Thomas Clusters with a 3x directionality. The rose diagrams for Thomas Clusters in **a)** are given in **d)**, with an IQR = 0.0006 with 2x directionality of **b)** given in **e)** with an IQR = 0.0007 and of 3x directionality in **f)** with an IQR = 0.0011. **g)** shows the isotropy of observed examples of a hydroid colony connected by stolon IQR = 0.0001, and in **h)** for the deep-sea sponge *Advhena magnifica* in two sections of a deep sea coral and sponge community subject to different current directions in **h)** IQR = 0.0023 and **i)** IQR = 0.0014 (from Ref. 24).

Figure 3: Boxplot of Isotropy as measured by the interquartile range (IQR) of point-to-point orientations grouped depending on whether they exhibit significant regularity. Lower IQR indicates isotropy, and higher IQR anisotropy. To visualise the IQR corresponds to directionality, rose diagrams for five example taxa are given on the right, with the corresponding IQR given on the boxplot in the same colour. The median percentile is shown, with the boxes providing the lower (25th) and upper (75th) quartiles and the whiskers are 1.5 times the interquartile range. Median Isotropy for non- regular taxa was 0.0009 and for regular spaced taxa 0.0027. Sample sizes are provided in Extended Data Table 1. We found an association between the IQR of cluster directionality (i.e. isotropy) and presence/absence of intra-specific regularity (Two-sided Mann Whitney $W = 25, N_1 = 10, N_2 = 11, p = 0.036$).

Figure 4: PCF for mapped taxa pairs that exhibit inter-specific segregation. For all plots the x-axis is the inter-point distance between organisms in meters. The y-axis PCF=1 indicate CSR, <1 indicates segregation, and >1 indicates positive association with the bars below the plots indicating whether the PCF is significantly > 1 (red), < 1 (blue) or CSR (grey). Heteromyopia is where the segregation (blue in the bottom bar) occurs at a smaller spatial scale (smaller x-axis value) in the inter-specific bar than in the univariate bars. **a)** Shows the spatial patterns for E surface *Primocandelabrum sp1.* and *Charniodiscus procerus*. **b)** Shows the spatial patterns for E surface *Fractofusus misrai* and *Charniodiscus procerus*. **c)** Shows the spatial patterns for E surface *Fractofusus misrai* and *Charniodiscus spinosus*. **d)** Shows the spatial patterns for Bed B *Charniodiscus sp1.* and *Charnia masoni*. **e)** Shows the spatial patterns for Lower Mistaken Point surface *Culmofrons plumosa* and Ostrich Feather.

Figure 5: Relationship between predictive variables with the most power and the minimum PCF. Black line shows the best fit for each regression is given and the grey area is the 95% confidence interval. **a)** is the regression between the IQR (cluster directionality) and the minimum PCF and **b)** between the percentage in the smallest size class (proxy for population age) and the minimum PCF.

Figure 6. Mechanistic model simulations with rejection sampling using ABCs with neural networks. **a)** Resulting alpha diversity from the 10,000 model simulations fitted to the observed (sampling corrected) alpha diversity for the Avalon, White Sea and Nama assemblages. The grey

dashed lines indicate the observed alpha diversity for the three assemblages whereby database occurrences were randomly subsampled to 50 occurrences from Ref. 23 The median percentile is shown, with the boxes providing the lower (25th) and upper (75th) quartiles and the whiskers are 1.5 times the interquartile range The median percentile is shown, with the boxes providing the lower (25th) and upper (75th) quartiles and the whiskers are 1.5 times the interquartile range. **b)** showing the posterior distribution for the dispersal parameter for the three assemblages, with low dispersal in the Avalon, high dispersal in the White Sea and Nama assemblages[12,40,73].

Line 41
Perhaps start a new paragraph here to guide the attention of the reader to the phenomenon of heteromyopia. In general, your paragraphs tend to be too long, please check throughout the text.

Thank you, we have implemented this change on Line 38

Figure 1
Figure 1 is a good idea, but panels a) and b) are of poor graphical quality due to the coarse dashed lines, and the arrows in panel c) are unclear. Perhaps the peak of the arrows should be placed at the centre of the corresponding black specimens. In panels a) and b), the dots should be larger, and all the circles should be solid.

Thank you, we have implemented your changes as shown below.

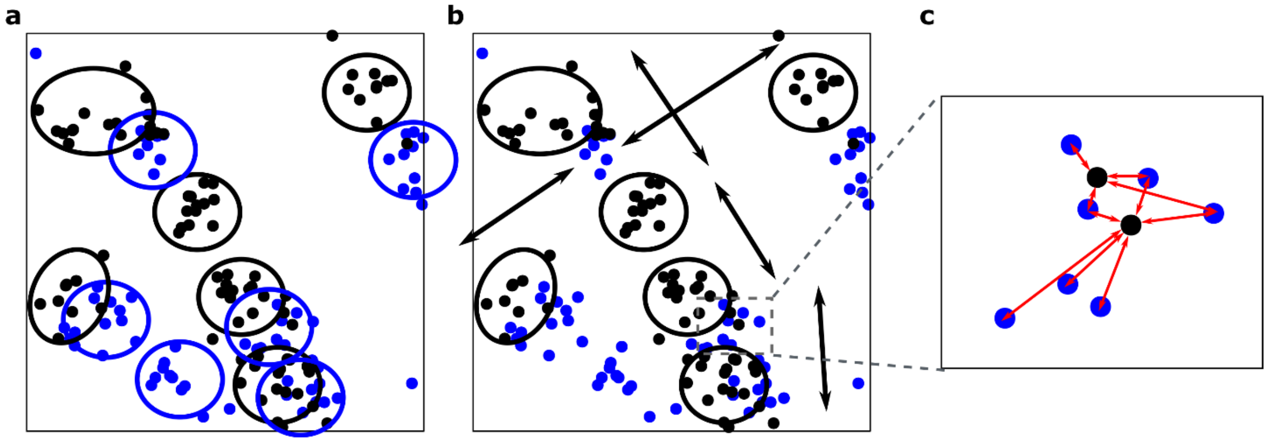

line 84
I would reduce the scope of this section to focus solely on the identification and quantification of stoloniferous reproduction, and open another section for the identification and quantification of resource competition

line 126
I would open here a new section "Identification and quantification of resource competition". This would make it easier to navigate through your manuscript.

We have implemented these changes on Lines 109 and 129.

Figure 2

The new figure 2 is nice, you could also provide the values of interquartile range for each isotropy plot. Additionally I would recommend the traditional way of a figure legend, explaining each panel a), b, ... i) individually in turn. You still can simplify e.g., e) same as d, but for the Thomas cluster process with a 2x directionality shown in a, f), ...

Generally, to help the reader, please label all panel with a, b, c, ... and refer then in the text to both the figure number and the associated panel label.

We have provided this information as given below on Lines 93.

Figure 2: Examples of different levels of isotropy. **a)** Simulated Thomas clusters with their associated rose diagram (as one would expect for clonal organisms)**. b)** Shows the same Thomas Clusters with a 2x directionality on the x-axis (as might be caused by broadcast spawning) and **c)** shows the same Thomas Clusters with a 3x directionality. The rose diagrams for Thomas Clusters in **a)** are given in **d)**, with an IQR = 0.0006 with 2x directionality of **b)** given in **e)** with an IQR = 0.0007 and of 3x directionality in **f)** with an IQR = 0.0011. **g)** shows the isotropy of observed examples of a hydroid colony connected by stolon IQR = 0.0001, and in **h)** for the deep-sea sponge *Advhena magnifica* in two sections of a deep sea coral and sponge community subject to different current directions in **h)** IQR = 0.0023 and **i)** IQR = 0.0014 (from Ref. 24).

line 106-113
It is unclear what your measure is. In general, the use of technical terms is often inconsistent. It would be very helpful for the reader if you could define a term unambiguously and then use it consistently for a given quantity. For example, you use 'rose diagram' versus 'isotropy plot', 'relative isotropy' versus 'isotropy' versus 'interquartile range' versus 'IQR', and so on.

Here you use all the terms "a metric to quantify cluster directionality", "relative isotropy", "interquartile range", "IQR", etc. to refer to the same thing. So, for example, this paragraph could read:
We used the interquartile range (IQR) of all point-to-point orientations within a population to quantify cluster directionality. The IQR was visualised by plotting the point-to-point orientation measurements as rose diagrams, where small values indicate isotropy (i.e. round isotropy plots; Fig. 2b, d and g) and high values indicate anisotropy (i.e. ovate isotropy plots; Fig. 2e, f, h and i).

Thank you for pointing out our inconsistency. We have been through the manuscript and ensured we also used IQR, rose diagram and isotropy throughout. We have implemented your suggestions to help clarify this point on Lines 107-116.

We used the interquartile range (IQR) of all point-to-point orientations within a population to quantify cluster directionality where the point-to-point orientations are the orientation between each point (here fossil specimen) to all the other points (conspecifics fossil specimens) within the population.This IQR visualised by plotting the point-to-point orientation measurements as rose diagrams, with isotropy indicated by small values, (i.e. round rose diagrams, Fig. 2 b,d,g), and anisotropy by high values, (i.e. ovate rose diagrams, Fig. 2e,f,h,i).

Line 113
I would start here a new paragraph, since you jump to a new issue (the 3 factors).

We have done so on Line 116

lines 128 – 145
Using the same terms for inter-specific and intra-specific measures is confusing. I would suggest making some small changes to your terminology to help readers recognise which analysis you are referring to. For intra-specific measures, you could use the established terms "spatial aggregation" (or simply "aggregation") and "regularity", and for inter-specific measures, you could use the terms "positive association" (or "attraction") and "segregation".

We have implemented the suggested changes throughout the manuscript.

line 137-145
You have to mention that you use the univariate pair correlation function for intra-specific analysis and the bivariate PCF for inter-specific analysis. In an intra-specific analysis, values below one corresponding to spatial regularity, above 1 to spatial aggregation. In an inter-specific analysis, values below one corresponding to spatial segregation, above 1 to spatial association (or attraction).

We have clarified this point as suggested on Lines 144-151:

We use univariate PCF for intra-specific analyses, where PCF = 1 represents complete spatial randomness, with values below one corresponding to spatial regularity, above 1 to spatial aggregation. For the inter-specific analyses PCF < 1 correspond to spatial segregation and PCF > 1 to positive spatial association. The strength of regularity/segregation, i.e. resource competition, is indicated by the smallest PCF value, i.e. the minimum value of the PCF, and the spatial scale over which resource competition occurs is the regularity/segregation up to (or over) the point where the observed data crosses PCF = 1[38].

Line 147
We started by applying our new directionality measure IQR to 21 (...)

Suggestion implemented on Line 154.

Line 148
What is "IQR", this is not previously defined in the text, but only in the figure 3 caption.

We have defined IQR on Lines 107-112.

We used the interquartile range (IQR) of all point-to-point orientations within a population to quantify cluster directionality where the point-to-point orientations are the orientation between each point (here fossil specimen) to all the other points (conspecifics fossil specimens) within the population. This IQR visualised by plotting the point-to-point orientation measurements as rose diagrams, with isotropy indicated by small values, (i.e. round rose diagrams, Fig. 2 b,d,g), and anisotropy by high values, (i.e. ovate rose diagrams, Fig. 2e,f,h,i).

line 171

“which would indicated effects of habitat associations” since you talk about an intra-specific analysis. Habitat segregation makes sense for two different species.

We have clarified this point on Lines 220-223:

There are ten taxa populations which had excursions under the Monte Carlo simulation envelopes that were not best modelled by heterogeneous Poisson models (which would indicate effects of habitat associations, cf. Ref. 10), so were considered to exhibit intra-specific competition….

lines 173, 174, 176, 197, 200, 208, etc
It is “regularity”, not “segregation”

These and all other intra-specific cases changed as suggested.

Figure 4
The idea of the figure is nice, but the figure design must be improved. The labels with the species names are far too small, and the coarse dashed lines should be solid lines with different colour to distinguish the different analyses. Each of the 5 cases should have an own label.

Thank you – we have checked minimum font size and increased our fonts, and also coloured in the lines. We haven’t made them solid to aid reading for colour-blind people.

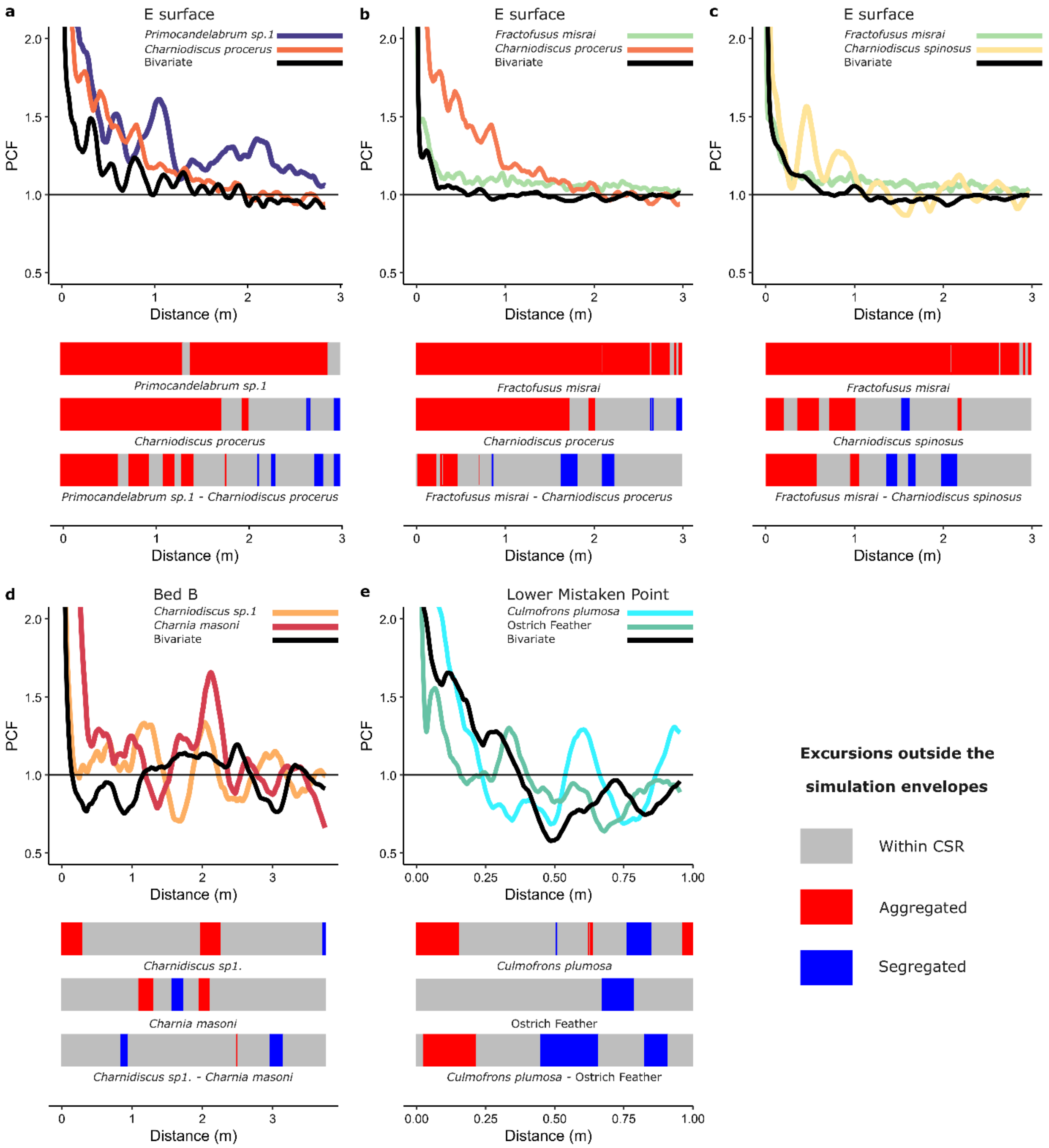

Figure 4: PCF for mapped taxa pairs that exhibit inter-specific segregation. For all plots the x-axis is the inter-point distance between organisms in meters. The y-axis PCF=1 indicate CSR, <1 indicates segregation, and >1 indicates positive association with the bars below the plots indicating whether the PCF is significantly > 1 (red), < 1 (blue) or CSR (grey). Heteromyopia is where the segregation (blue in the bottom bar) occurs at a smaller spatial scale (smaller x-axis value) in the inter-specific bar than in the univariate bars. **a)** Shows the spatial patterns for E surface *Primocandelabrum sp1.* and *Charniodiscus procerus*. **b)** Shows the spatial patterns for E surface *Fractofusus misrai* and *Charniodiscus procerus*. **c)** Shows the spatial patterns for E surface *Fractofusus misrai* and *Charniodiscus spinosus*. **d)** Shows the spatial patterns for Bed B

Charniodiscus sp1. and *Charnia masoni*. **e)** Shows the spatial patterns for Lower Mistaken Point surface *Culmofrons plumosa* and Ostrich Feather.

line 196
Introduce a new section "Relationship between stoloniferous reproduction and strength of intra-specific regularity"

Introduced on Line 203

line 200
It should be Fig 5 I guess.

We are referring to the boxplot, not the regression plot, so Fig. 3 is correct.

lines 207-212, 137
Ok, use of the pair correlation functions is fine. You showed that the results are insensitive to the bandwidth (and distance range), which is important indication of robustness.

Thank you, we very much appreciate the opportunity to demonstrate the robustness of our results.

line 209
Extended Data Table 4 I guess?

Yes, thank you for catching

Figure 5
The new Figure 5 is also a nice addition! It is a good idea to brake it down in terms of predictor variables.

Thank you.

line 262
Introduce a new paragraph

lines 272-297
Too long paragraph, split

line 298
Add a new section e.g., "A mechanistic model to investigate the effect of dispersal limitation" to help navigation through your paper.

line 306
Introduce a new paragraph to allow the reader to quickly find the model description.

We have implemented these suggestions on Lines 273, 292, 307 and 317.

line 306-317
Ok, it is fine that the mechanistic model includes heteromyopia only indirectly via dispersal

limitation. However, you should state explicitly in the text that heteromyopia is in the model a consequence of high dispersal limitation, so that the reader can re-connect with the arguments in the summary and the introduction, as well with the results of the spatial analysis.

Thank you, we have added this suggestion on Lines 327-328:

Within this model, heteromyopia is a consequence of high dispersal limitation.

lines 673
I am also happy with the additional explanation of the edge correction method and the Thomas cluster methods. But start with a new paragraph.

Thank you, implemented on Line 424

line 719
Add "describing 100 communities on a grid network."

We have done so on lines 469-470:

… diversity, we use the metacommunity model of Ref. 67 describing 100 communities on a grid network.

Line 739
The description of spatial autocorrelation is confusing. Maybe just say that high autocorrelation means that communities closes to each other have similar environmental conditions, whereas low autocorrelation means that the environmental conditions of nearby communities are uncorrelated.

Thank you, we have clarified this text on Lines 489-493:

Spatial autocorrelation defined as high autocorrelation reflecting communities that are closer together have similar environmental conditions, whereas low spatial autocorrelation means that the environmental conditions of nearby communities are unconnected. Formally, spatial autocorrelation is defined in this model as the inverse in the sum of the differences in the environmental conditions, between each pair of connected communities.
